# Effect of a Gamified Family-Based Exercise Intervention on Adherence to 24-Hour Movement Behavior Recommendations in Preschool Children: Single-Center Pragmatic Trial

**DOI:** 10.2196/60185

**Published:** 2025-03-04

**Authors:** Gaizka Legarra-Gorgoñon, Yesenia García-Alonso, Robinson Ramírez-Vélez, Loreto Alonso-Martínez, Mikel Izquierdo, Alicia M Alonso-Martínez

**Affiliations:** 1 Navarrabiomed, Hospital Universitario de Navarra, Universidad Pública de Navarra, Instituto de Investigación Sanitaria de Navarra, Avda Barañain s/n, Pamplona, 31008, Spain, 34 948169000; 2 Biomedical Research Center (CIBER) of Frailty and Healthy Aging, Instituto de Salud Carlos III, Madrid, Spain

**Keywords:** children, gamification, exercise, physical fitness, domains of physical activity, game, fitness, child, family-based, exercise program, randomized controlled trial, strength

## Abstract

**Background:**

Adherence to 24-hour movement behavior recommendations, including physical activity (PA), sedentary time, and sleep, is essential for the healthy development of preschool children. Gamified family-based interventions have shown the potential to improve adherence to these guidelines, but evidence of their effectiveness among children is limited.

**Objective:**

This study aimed to evaluate the effectiveness of a gamified family-based exercise intervention in promoting adherence to 24-hour movement behavior recommendations among preschool-aged children.

**Methods:**

This 12-week study is a single-center, pragmatic randomized controlled trial that included 80 preschool children (56% boys) and their families, who were randomly assigned to either the gamification group (n=40) or the control group (n=40). The “3, 2, 1 Move on Study” incorporates family-oriented physical activities and gamification techniques to increase PA domains, reduce sedentary behavior, and improve sleep patterns. The primary outcome was to increase moderate to vigorous PA (MVPA) by 5 minutes/day, as measured by accelerometer at follow-up. Accelerometer-determined daily time spent (PA domains, sedentary behavior, and sleep), physical fitness (cardiorespiratory, speed-agility, muscular, physical fitness z-score), basic motor competencies (self-movement and object movement), and executive function (memory, cognitive flexibility, and inhibitory control) were also included as secondary outcomes.

**Results:**

The 71 participants included in the per-protocol analyses (32 girls, 45%; 39 boys, 55%) had a mean (SD) age of 5.0 (0.5) years. Change in MVPA per day after the intervention (12 weeks) increased in both groups by +25.3 (SD 24.6) minutes/day in the gamification group and +10.0 (SD 31.4) minutes/day in the routine care group, but no significant between-group differences were observed (8.62, 95% CI –5.72 to 22.95 minutes/day, *ηp*^2^=.025; *P*=.23). The analysis of secondary outcomes showed significant between-group mean differences in the change in physical behaviors derived from the accelerometers from baseline to follow-up of 26.44 (95% CI 8.93 to 43.94) minutes/day in favor of light PA (*ηp*^2^=.138; *P*=.01) and 30.88 (95% CI 4.36 to 57.41) minutes/day in favor of total PA, which corresponds to a large effect size (*ηp*^2^=.087; *P*=.02). Likewise, the gamification group substantially increased their score in standing long jump and physical fitness z-score from baseline (*P*<.05).

**Conclusions:**

In the “3, 2, 1 Move on Study,” a gamified intervention showed a modest but relevant increase in MVPA and other domains of 24-hour movement behavior among preschool-aged children. Therefore, gamified family-based interventions may provide a viable alternative to improve adherence to 24-hour movement behavior recommendations.

## Introduction

Physical activity (PA) is essential for early childhood development [1] and positively impacts skeletal growth [2], metabolism [3], and cardiovascular health [4]. At the psychological level, PA has been shown to enhance self-esteem [5], reduce stress [6], and improve social behaviors. In 2019, the World Health Organization (WHO) issued guidelines for children under 5 years of age. These underlined the importance of the early habits of PA adoption for health promotion and prevention of obesity throughout life. For children aged 3‐4 years, the WHO recommends at least 180 minutes of daily PA, including 60 minutes of moderate to vigorous intensity, to support physical, cognitive, and motor development [7]. Additionally, children should not remain sedentary for more than an hour at a time, screen time should be limited to less than 1 hour per day, and they should get 10‐13 hours of sleep with regular sleep and wake times [7]. Preschool-aged children have a unique need to establish healthy activity patterns, as they rely on structured, supervised activities. Effective interventions for this age group should offer engaging and developmentally appropriate activities that encourage movement and active play.

Adherence to these PA recommendations is associated with improved mental health outcomes, including reduced symptoms of anxiety and depression [8,9] and the promotion of healthier lifestyles [10]. However, an upward trend in childhood sedentarism has been observed in recent decades [11]. A systematic review by Tucker et al [12] reported that only 54% of children aged 2‐6 years met the PA guidelines of the National Association of Sport Officials. Research further indicates a decline in PA from early childhood to adolescence [13,14], which contributes to the increased rates of overweight and obesity in this population, as well as a decline in cardiorespiratory fitness (CRF) [15,16].

Physical inactivity and sedentary behavior (SB) have contributed to declining physical fitness (PF) levels among children [17]. Given the established health benefits of engaging in regular moderate-to-high-intensity PA, this level of PA is recommended to support improvements in PF components, including CRF, musculoskeletal fitness (muscle strength and endurance), and flexibility [18]. By emphasizing these intensity levels, one can maximize their potential fitness gains to help with overall health and functional capacity [19].

PA is also important for the development of motor skills in young children because it provides the practice and experience needed to improve coordination, balance, agility, and other essential motor competencies. Indeed, previous cross-sectional studies have noted a positive relationship between PA and PF in preschoolers, with higher PA levels also linked to superior motor skills performance [20]. Motor skill competency is considered an integral part of PF in children since it enables them to successfully engage in various activities—skills that form the foundation for lifelong active habits. The development of these motor skills tends to contribute to overall PF by enhancing cardiovascular health, muscular strength, and endurance; increasing a child’s confidence and enjoyment of physical activities; and encouraging the development of positive attitudes toward regular PA. In this study, we considered motor skill competency as an integral part of PF, emphasizing the importance of early interventions targeting PA levels and motor development to promote holistic PF outcomes in preschool-aged children [21].

Gamification techniques, including badges, leaderboards, streaks, team-based competition, and story elements, have been proven to improve many health behaviors beyond weight loss [22]. Specifically, mobile health interventions using “exergames” have successfully embedded gamified elements to enhance enjoyment and increase engagement in PA [23-25]. Evidence on younger populations has shown that gamified interventions have the potential to enhance PA levels, motor skills, social interaction, and emotional well-being [26]. For example, virtual reality combined with gamification in PA programs has been reported to enhance motivation, thereby increasing the extent of engagement in exercise [27]. In adolescents, gamification particularly enhances motivation and adherence to PA via social or competitive elements appealing to this phase of development [28]. These results emphasize the need for age-appropriate gamified interventions that allow each age group to engage optimally and achieve even better improvements in PA levels and health outcomes. To date, few attempts to gamify weight loss have prioritized increasing PA, and they have shown only modest success [22]. Thus, gamification strategies can bolster health habits and promote appropriate PA to inspire behavioral changes through enhanced motivation [29]. In this context, Sailer et al [30] posit that gamification can potentially improve motivational challenges while being supported by robust implementation models.

Therefore, integrating motivational tactics is likely to positively affect PA behaviors and the related motivational outcomes. To better understand under which circumstances gamification improves adherence to 24-hour movement behavior recommendations, for whom the effects would be most beneficial, and which aspects of physical health would be best targeted, we designed a nature-based intervention, the “3, 2, 1 Move on Study” trial, consisting of gamified training for 15-37 minutes per week for 12 weeks. We hypothesized that the “3, 2, 1 Move on Study” would lead to improved PA levels, reduced SB, and enhanced sleep patterns.

## Methods

### Study Design and Participants

The “3, 2, 1 Move on Study” is a single-center, pragmatic randomized controlled trial (registered on ClinicialTrials.gov; NCT05741879) of 12 weeks of gamified training from November 2022 to February 2023, delivered through Iturrama Primary Care Center (Pamplona, Spain).

**Figure 1.**
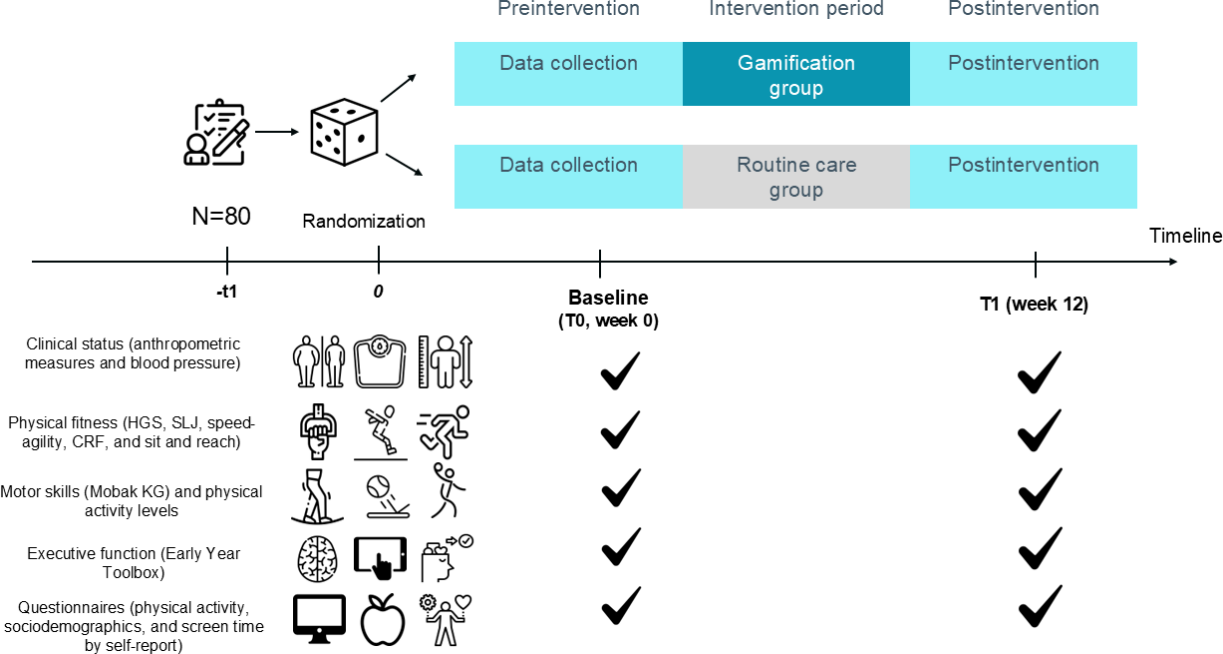
Study design of the “3, 2, 1 Move on Study.” Participating children were randomly assigned to either the 12 weeks of routine care group (n=40) or the gamification group (n=40). CRF: cardiorespiratory fitness; HGS: handgrip strength; SLJ: standing long jump.

This trial followed the CONSORT (Consolidated Standards of Reporting Trials) reporting guideline, including reporting of protocol deviations [31]. The overall study procedure is illustrated in Figure 1.

A brief description of the methodology was peer reviewed and published [31]. All participants were from the Iturrama Primary Care Center (Pamplona, Spain). The parents or legal guardians of the children provided written informed consent to participate in the trial. Children must not have a diagnosis of any musculoskeletal, cardiovascular, pulmonary, or orthopedic problems; disabilities precluding the participant from being physically active; or any other physical condition that precludes the participant from being physically active. Children who did not comply with established procedures and those who did not understand Spanish were excluded from the study. Throughout this protocol, the trial staff was responsible for weekly monitoring of various aspects, including meetings discussing topics related to managing the platform and performing exercises. Additionally, dedicated email addresses and phone numbers were provided to address any questions or concerns.

### Ethical Considerations

The study was conducted in accordance with the Declaration of Helsinki and approved by the Clinical Research Ethics Committee of Navarra (PI_2021/111). Participation of children was voluntary, and their parents provided written informed consent. No compensation was provided to participants.

### Randomization

The participants were randomly assigned to 1 of the 2 groups before the commencement of the study. The stratified randomization (1:1 balance between the number of children in the gamification and control groups) was computerized by an independent, blinded statistician not involved in the study (Research Randomizer V.4). Allocation assignment was concealed to the researchers at the analysis stage.

### Intervention and Control

A family-based, gamified intervention was developed by experts in education and sport sciences to create a structured PA program specifically designed for preschool children. This approach emphasizes the use of playfulness, which is important to engage young children, while integrating game mechanics such as point systems, rewards, and progress tracking. These elements were strategically implemented to enhance motivation and promote adherence to the PA program. The intervention activities incorporated customizable features, including adjustable difficulty levels and personalized avatars, to sustain participants’ interest and engagement. Family involvement was emphasized as a core component, recognizing that young children often require adult support and encouragement to fully engage in the program. The intervention design was based on the premise that gamified family-centered activities can establish a supportive setting, thereby making PA both enjoyable and sustainable for children and their parents. Participants in this group were invited to a 12-week exercise program on an online gamified platform, with the idea of encouraging children to engage in PA with their families either at home or in an outdoor space. Each child had a user account to log in and complete exercises twice per week, with one session available from Monday to Wednesday and a repeat session available from Thursday to Sunday. The program comprised 3 phases: a warm-up/activation phase, a training phase (featuring body weight strength exercises, CRF exercises, and color-coded exercises), and a cooldown phase. The number of exercises and repetitions varied from week to week. The warm-up phase consisted of 3 minutes of continuous light-intensity training, which lasted 10‐26 minutes with 6‐12 repetitions, 20‐40 seconds of work, and 10‐20 seconds of recovery. The cooldown phase involved 3 minutes of static and dynamic exercises. Exercises were changed weekly, gradually increasing in length and intensity. The parents or guardians of the children supervised the program.

**Figure 2.**
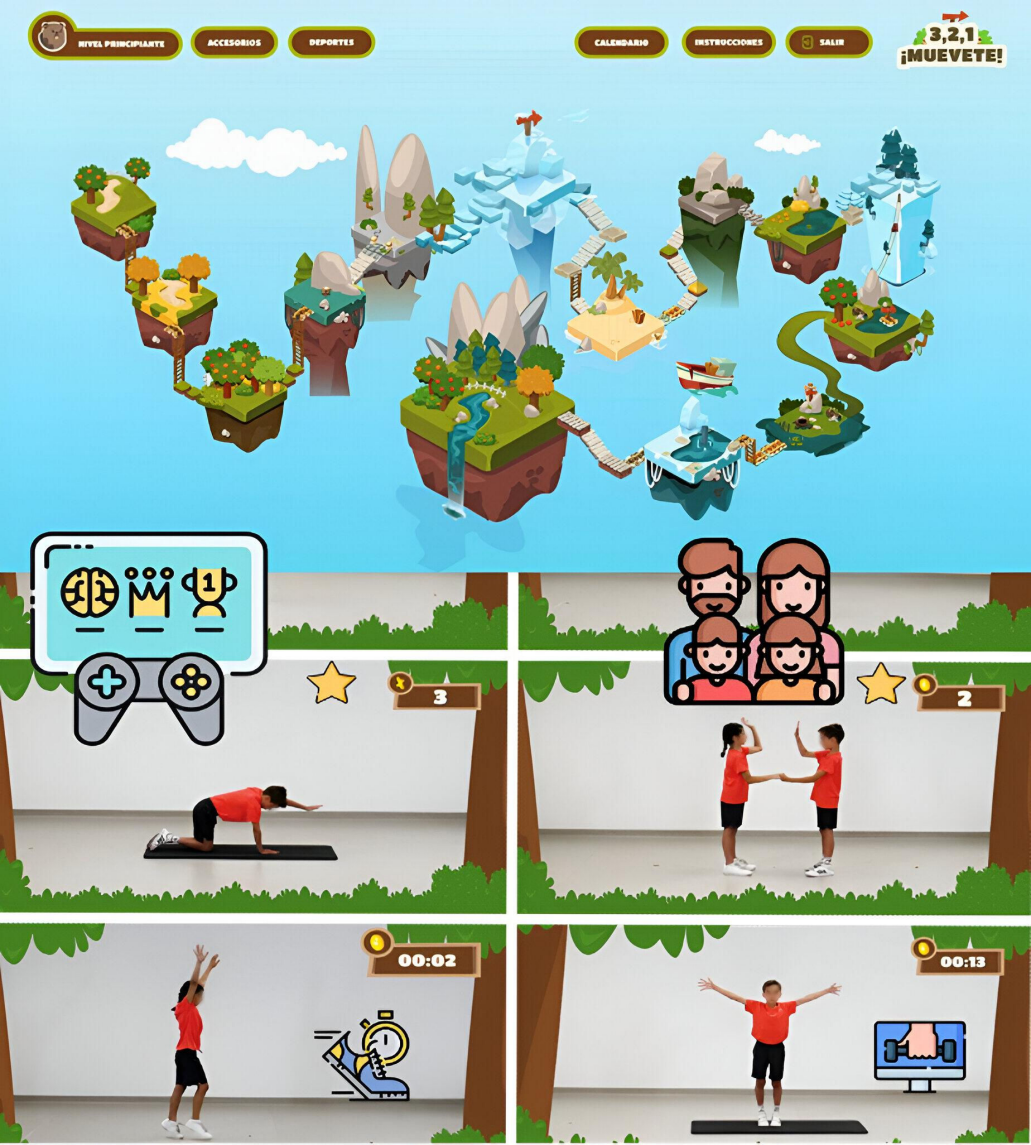
Framework screen of the gamified online platform.

Figure 2 illustrates the main screen of the platform viewed by participants upon completing each session. At the beginning of the game, the children were prompted to select and customize their avatars by entering their name, choosing the avatar type, and customizing its appearance based on their preferences. The personalization feature was designed to instill a sense of ownership and, consequently, enhance participant engagement. As participants progressed through the program, they unlocked new “worlds” and earned additional rewards. The platform consisted of 14 worlds, with each milestone providing opportunities to access new accessories and further personalize avatars (after completing worlds 3, 6, 9, and 12). This reward system was designed to maintain interest and provide continuous progress reinforcement, which is crucial for sustained motivation among younger users.

Children advanced through a structured level system, progressing from the “novice level” to the “expert level.” This structure ensured they were consistently challenged in line with their developmental skills while fostering a systematic sense of progress. The platform also featured a calendar that guided participants on the appropriate session or world to engage with, based on the program’s week. This scheduling tool facilitated participants in organizing their training progression, establishing clear and attainable goals, and monitoring their improvement over time. Overall, the gamified platform integrated PA with an engaging and dynamic experience, encouraging children to remain active while enjoying the process.

To increase motivation and adherence, the research team was responsible for ensuring participants’ compliance with the study requirements by supervising their participation in 2 weekly sessions. Each week, access to the exercise platform was monitored by evaluating the total usage time per session and verifying the completion of the prescribed training program. Participants who did not adhere to these guidelines were contacted via telephone to identify the reasons for noncompliance and to provide encouragement and support to continue their participation.

The control group continued with usual routine care according to the international guidelines set by the WHO [7]. Activities included regular physical education classes at school and opportunities to play with friends at parks or in backyards. The parents or guardians in the control group were not prohibited from noting the children’s PA levels on their own.

### Outcome Measures

The primary outcome was a change in the child’s 24-hour movement behavior recommendations between baseline and 12 weeks (increasing PA levels, reducing SB, and enhancing sleep patterns) as measured by the GENEActiv Original triaxial accelerometer, with a frequency of 87.5 Hz, over 7 consecutive days on the nondominant wrist to record PA and sleep information. Specifically, the “3, 2, 1 Move on Study” will be able to detect a difference in the mean daily MVPA between the gamification group and routine care group of ±5 minutes at follow-up. Activity patterns at baseline and during the intervention were collected through GENEActiv PC software (version 3.3) and processed and analyzed using the R package GGIR as described elsewhere [32]. Briefly, thresholds for children aged 4‐6 years were as follows: <56.3 (mg) for sedentary time, ±56.3 mg light PA (LPA), 191.6 mg moderate PA (MPA), and ≥695.8 mg vigorous PA (VPA). Sleep patterns were calculated using the algorithm developed by van Hees et al [33].

Secondary outcomes were changes in health-related PF as measured by the PREFIT battery [34]. Participants completed each test consecutively, except for the 20-meter shuttle run, where multiple children ran simultaneously in small groups. An analog handgrip dynamometer was used to measure upper body muscular strength (grip strength Dynamometer T.K.K. 5001 Grip). The handgrip strength (HGS) test was performed twice for each hand, where the children gradually squeezed the dynamometer for 2‐3 seconds. The highest value for each hand was recorded, and the average of these 2 results was calculated to analyze the upper-body muscular strength measurements. The standing long jump (SLJ) test was used to assess lower body strength. The children performed a maximal horizontal jump from a standing position, landing on both feet, while maintaining an upright position. The test was conducted 3 times, and the best result, in centimeters, was recorded. Motor competence was assessed using the 4 × 10-meter shuttle run test of speed of movement, agility, and coordination. Lower scores indicated better performance (in seconds) and these were recorded for analysis. CRF was assessed using the 20-meter shuttle run (laps and estimated maximum oxygen consumption). An incremental audio signal was used, starting at 6.5 km/h and increasing by 0.5 km/h per minute. The test ended when the child failed to reach one of the lines on 2 consecutive occasions with the audio signal or when the child stopped owing to exhaustion [35]. Finally, a previously validated overall PF score was calculated [36]. The individual score of each PF component was transformed into sex-specific standardized values (z-scores). An overall PF z-score was calculated as the mean of the z-score values for HGS, SLJ, the 4 × 10-meter shuttle run test (for analytic purposes, values were multiplied by −1, so higher scores indicate better motor competence), and the CRF test. Higher z-score values in PF indicate better fitness performance.

The MOBAK KG test battery was used to evaluate basic motor competencies (BMC) in preschool children aged 4‐6 years [37]. This assessment comprises 8 test items to measure 2 key domains: self-movement (SM: balancing, rolling, jumping, running) and object movement (OM: throwing, catching, bouncing, dribbling). Each domain includes 4 items, with a maximum score of 8 points per domain, resulting in a total possible score of 16 points (highest BMC). Children were not provided with practice attempts before the assessment. For the “throwing” and “catching” tasks, children had 6 attempts, with scoring based on success rates: 0 points for 0‐2 successful attempts, 1 point for 3‐4 attempts, and 2 points for 5‐6 attempts. In the “bouncing,” “dribbling,” “balancing,” “rolling,” “jumping,” and “running” tasks, participants had 2 attempts each, scored dichotomously: 0 points for no success, 1 point for one success, and 2 points for two successful attempts.

Executive function was evaluated using a digital testing platform on iPads, administered by trained research assistants. The assessment incorporated tasks from “Early Tools” (Years Toolbox YET-2017), including “Mr. Ant” and “Not This” to assess memory, “Card Sorting” to measure cognitive flexibility, and “Go/No-Go” to evaluate inhibitory control [38]. These tasks are designed to provide a comprehensive profile of executive function in children.

Anthropometric measurements were taken to determine the weight (kg), height (cm), waist circumference (WC), and BMI of the children. These assessments were conducted following the protocol established by the Centers for Disease Control and Prevention’s National Health and Nutrition Examination Survey (CDC-NHANES), with trained evaluators performing the measurements [39]. Height was measured using a stadiometer with an accuracy of 1 mm (SECA 213 model from Seca Ltd) in the Frankfurt position. Weight was measured using a Tanita DC-430 body composition analyzer with an accuracy of 100 g (Tanita DC430 MA model, Tanita Corporation). BMI was calculated by dividing weight (kg) by the square of height (m). WC was measured twice to the nearest 1 mm with a nonelastic tape applied horizontally midway between the lowest rib margin and the iliac crest, at the end of gentle expiration with the children in a standing position. Triceps thickness (mm) was measured twice on the right side of the body to the nearest 0.2 mm with a skinfold calliper (Holtain, range 0‐40 mm, Holtain Ltd) halfway between the acromion and the olecranon process at the back of the arm. A validated automatic oscillometric device (Omron 705-IT) was used to assess blood pressure. Last, a questionnaire including self-reported variables was completed by the participants’ parent(s) or guardian(s) via Google Forms. This questionnaire will include information about the child as well as details about the parent(s) or guardian(s), such as maternal and paternal education, socioeconomic status, or clinical child data records.

### Statistical Analysis

Descriptive sample characteristics were presented as mean (SD) or frequency (percentage). All variables were checked for normality using both graphical (normal probability plots) and statistical (Kolmogorov–Smirnov test) procedures. We conducted per-protocol analyses using a repeated measures ANOVA to test for significant differences across the time points. The interaction effect between group and time was assessed using repeated measures analysis of covariance (ANCOVA), with the baseline as the covariate. To describe the differences in the related treatments, the effect size between-group differences were calculated using the partial eta squared (*η*_p_^2^), which was interpreted considering the *η*_p_^2^ values of 0.01, 0.06, and 0.14, which correspond to small, moderate, and large effect sizes, respectively [40]. Statistical analyses were conducted using IBM SPSS statistical software (version 26.0; IBM Corp). The level of statistical significance was set at *P*<.05.

### Protocol Deviations

There were several protocol deviations from the trial registration. First, we planned to exclusively recruit from a list of 120 participants enrolled in a cross-sectional study[31]. We began the recruitment process using this list, but responses were substantially lower than anticipated. Second, the 24-week follow-up could not be conducted due to many parents reporting challenges in coordinating the follow-up appointments because of their work and personal commitments. Other common reasons that parents declined to participate included (1) not wanting to add strain to the curriculum because of a lack of time at the end of the course, (2) difficulty obtaining parental consent forms, and (3) not wanting to add to their workload. Third, some families had difficulties maintaining consistent communication. Although parental involvement was crucial for achieving a high level of compliance with the intervention, parents and children in this study may represent a subgroup with a particularly high motivation to reduce screen media use, as families volunteered to participate, which could influence the generalizability of the results at 24-week follow-up. Finally, the response rate to the questionnaires was unexpectedly low, leading to incomplete data collection and posing a challenge to acquiring reliable information within the intended time frame.

## Results

### Participants

**Figure 3.**
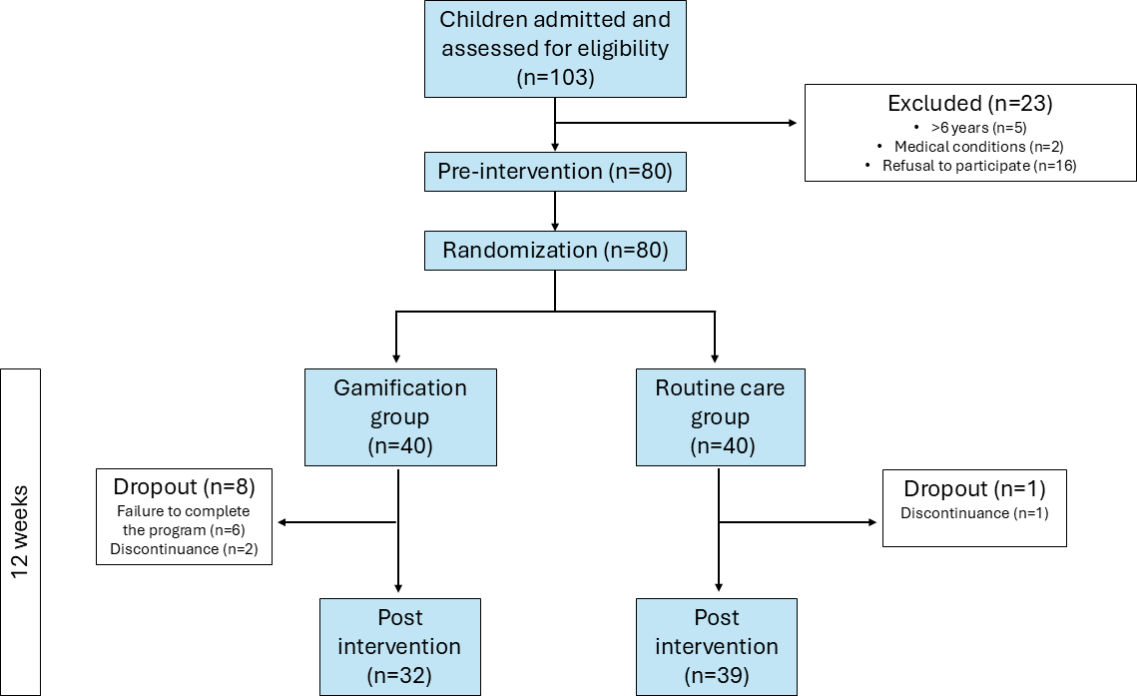
CONSORT flow diagram. CONSORT: Consolidated Standards of Reporting Trials.

The flow of participants through the trial is described in Figure 3, showing that 40 children were randomly assigned to the routine care group and 40 were assigned to the gamification group. After 12 weeks of intervention, 32 and 39 children in the gamification and routine care groups, respectively, were assessed. A total of 32 participants completed the gamification sessions. Among them, 28 achieved an adherence rate of over 70% to the exercise sessions, while the remaining 4 demonstrated adherence above 50%. A total of 8 dropouts were recorded in the gamification group because they failed to meet the minimum number of sessions required. One dropout occurred in the routine care group due to a change in the primary care center during the intervention.

The baseline characteristics of the participants are presented in Table 1. Among the children, most were boys (n=44, 55%) and aged from 4.1 to 5.7 years, with an average age of 5.0 (SD 0.5) years. Baseline characteristics were generally similar across the 2 groups.

**Table 1.** Participant characteristics at baseline. Data are presented as mean (SD) or frequency (percentage).

Characteristics	Gamification group (n=40)	Routine care group (n=40)	Full sample (n=80)
Sex (boys/girls), n	18/22	26/14	44/36
Age (years), mean (SD)	5.1 (0.5)	4.9 (0.5)	5.0 (0.5)
Height (cm), mean (SD)	110.3 (5.3)	109.3 (5.1)	109.8 (5.2)
Weight (kg), mean (SD)	20.0 (3.6)	19.4 (4.0)	19.7 (3.8)
BMI (kg/m^2^), mean (SD)	16.4 (1.8)	16.1 (2.4)	16.2 (2.1)
Waist circumference (cm), mean (SD)	53.8 (4.6)	54.0 (2.4)	53.9 (5.6)
Fat mass (%), mean (SD)	21.5 (4.3)	20.8 (4.7)	21.1 (4.5)
Lean mass (%), mean (SD)	73.8 (4.1)	74.5 (4.4)	74.2 (4.2)
Triceps skinfold thickness (mm), mean (SD)	12.9 (3.8)	13.8 (4.1)	13.3 (4.0)
Systolic blood pressure (mm Hg), mean (SD)	97.9 (8.8)	99.5 (6.2)	98.7 (7.6)
Diastolic blood pressure (mm Hg), mean (SD)	60.8 (7.0)	63.8 (7.4)	62.3 (7.3)
Maternal university education, %	66	82	74
Paternal university education, %	40	43	42
Monthly family income ≥€3000 (≥US $3146.43), %	50	70	60

### Outcomes

Table 2 lists the effects of the groups on primary and secondary outcomes. In per-protocol analyses, the primary outcome (mean minutes change in MVPA per day, after the 12-week intervention) increased in both groups, by +25.3 (SD 24.6) minutes/day in the gamification group, and +10.0 (SD 31.4) minutes/day in the routine care group, but no significant between-group differences were observed (8.62, 95% CI –5.72 to 22.95 minutes/day; *ηp*^2^=.025; *P*=.23). The analysis of secondary outcomes showed significant between-group mean differences in the change in physical behaviors derived from the accelerometers from baseline to follow-up of 26.44 (95% CI, 8.93 to 43.94) minutes/day in favor of LPA (*ηp*^2^=.138; *P*=.004) and 30.88 (95% CI 4.36 to 57.41) minutes/day in favor of TPA, which corresponds to a large effect size (*ηp*^2^=.087; *P*=.02). Likewise, the gamification group substantially increased their score on the SLJ and overall PF (z-score) from baseline, with a large effect size between groups for the SLJ of +12.26 (95% CI 6.23 to 18.28) cm (*ηp*^2^=.205; *P*<.001) and overall PF by 0.15 (95% CI 0.01 to 0.29) z-score (*ηp*^2^=.076; *P*=.04). No significant differences between groups were observed in the change in other 24-hour movement behavior composition (MPA, VPA, SB, sleep times), PF domains (HGS, 4 × 10-meter test), or BMC outcomes. Similarly, per-protocol analyses showed no effects on executive function scores (Table 2).

**Table 2.** Per-protocol analysis of primary and secondary outcomes at baseline and changes after 12 weeks.

Outcome		Groups				Within-group difference		Between-group difference	
		Baseline, mean (SD)		Follow-up, mean (SD)		Mean differences (SD)		Mean differences (95% CI)	
		Gamification (n=40)	Routinecare (n=40)	Gamification (n=32)	Routinecare (n=39)	Gamification	Routinecare	Gamification online minus routine care	*ηp*^2^ (*P* value)
**Primary outcome**									
	MVPA^a^ (min/day)	66.8 (25.1)	79.7 (32.4)	89.9 (33.9)	90.4 (30.6)	25.3 (24.6)	10.0 (31.4)	8.62 (–5.72 to 22.95)	.025 (.23)
**Secondary outcomes**									
	LPA^b^ (min/day)	242.7 (40.6)	249.1 (36.7)	269 (34.5)	253.6 (41.3)	33.4 (33.3)	0.3 (39.9)	26.44 (8.93 to 43.94)	.138 (.01)
	MPA^c^ (min/day)	57.8 (20.1)	68.5 (26.4)	76.7 (25.8)	75.7 (23.4)	21.1 (19.3)	6.5 (24.5)	8.46 (–2.57 to 19.50)	.040 (.13)
	VPA^d^ (min/day)	8.9 (5.8)	11.2 (6.6)	13.2 (8.7)	14.7 (8.8)	4.1 (5.8)	3.5 (7.8)	0.20 (–3.47 to 3.88)	.000 (.91)
	TPA^e^ (min/day)	309.5 (56.8)	328.8 (62.9)	358.9 (50.6)	344.1 (5.0)	58.7 (49.7)	10.4 (64.4)	30.88 (4.36 to 57.41)	.087 (.02)
	SB^f^ (min/day)	612.5 (87.0)	599.2 (78.2)	550.9 (66.5)	559.5 (71.4)	–69.8 (82.1)	–40.9 (76.6)	–13.61 (–45.98 to 18.76)	.012 (.40)
	Sleep (hours/day)	8.6 (0.9)	8.5 (0.8)	8.8 (0.6)	8.9 (0.6)	0.2 (0.9)	0.5 (0.8)	–0.22 (–0.49 to 0.06)	.043 (.12)
	HGS^g^ (kg)	7.4 (2.1)	8.0 (2.1)	7.4 (2.0)	8.0 (2.3)	0.3 (2.4)	–0.1 (1.8)	–0.80 (–1.69 to 0.09)	.000 (.90)
	SLJ^h^ (cm)	87.6 (21.8)	98.2 (15.3)	99.0 (23.0)	93.9 (14.4)	10.8 (10.8)	–4.2 (14.3)	12.26 (6.23 to 18.28)	.205 (<.001)
	4 × 10-meter test (s)	16.0 (2.0)	15.5 (1.6)	15.2 (1.8)	15.1 (1.2)	–1.0 (1.4)	–0.4 (1.2)	–0.34 (–0.87 to 0.19)	.027 (.20)
	CRF^i^ (laps)	24.2 (10.7)	22.4 (10.8)	25.6 (11.8)	24.0 (11.1)	1.4 (5.9)	2.1 (9.4)	0.29 (–3.61 to 4.20)	.000 (.88)
	PF^j^ (z-score)	–0.0 (0.5)	0.0 (0.4)	0.0 (0.4)	0.0 (0.4)	0.1 (0.3)	–0.1 (0.3)	0.15 (0.01 to 0.29)	.076 (.04)
	Mobak OM^k^ (points)	2.6 (1.9)	3.2 (2.23)	3.4 (2.4)	3.5 (2.3)	0.7 (1.9)	0.3 (1.7)	0.24 (–0.61 to 1.09)	.005 (.57)
	Mobak SM^l^ (points)	3.9 (2.4)	3.7 (2.4)	4.5 (2.2)	3.8 (2.3)	0.2 (1.8)	0.1 (1.8)	0.27 (–0.78 to 1.33)	.003 (.67)
	Mobak total (points)	6.4 (3.8)	6.9 (3.8)	7.6 (4.2)	7.3 (4.1)	0.9 (2.9)	0.4 (2.2)	0.42 (–0.80 to 1.64)	.007 (.49)
	Mr. Ant	2.3 (0.9)	2.0 (0.8)	2.7 (1.4)	2.2 (0.9)	0.5 (1.6)	0.2 (0.8)	0.46 (–0.24 to 1.16)	.036 (.19)
	Go/no-go	0.8 (0.2)	0.7 (0.2)	0.8 (0.1)	0.8 (0.2)	–0.0 (0.2)	–0.0 (0.2)	0.02 (–0.07 to 0.10)	.311 (.73)
	Not this	2.5 (0.6)	2.5 (0.7)	2.6 (0.7)	2.6 (0.8)	0.1 (0.8)	0.3 (0.8)	–0.13 (–0.55 to 0.28)	.003 (.92)
	Card sorting	8.5 (3.0)	7.8 (3.5)	9.1 (2.7)	8.2 (2.8)	1.0 (2.3)	–0.2 (2.4)	1.08 (–0.16 to 2.32)	.066 (.08)

a MVPA: moderate to vigorous physical activity.

b LPA: light physical activity.

c MPA: moderate physical activity.

d VPA: vigorous physical activity.

e TPA: total physical activity.

f SB: sedentary behavior.

g HGS: handgrip strength.

h SLJ: standing long jump.

i CRF: cardiorespiratory fitness.

j PF: physical fitness.

k OM: object movement.

l SM: self-movement.

## Discussion

### Principal Findings

The major findings of this study showed that a gamified family-based exercise could be an effective intervention to affect preschool children’s adherence to 24-hour movement behavior guidelines. The intervention successfully increased PA levels and decreased SB within the target population. In fact, this program optimized both engagement and adherence through gamification, hence proving again that young children enjoy active play and interactive approaches. Point accumulation, immediate rewards, and progress tracking were some of the features integrated to enhance positive behaviors and encourage consistent participation. The program also provided a supportive environment where the inclusion of family members in the process of developing healthier habits should help in reinforcing bonds between family members. This novel approach encouraged participation and also supported the establishment of lifelong PA habits in young children, which is crucial for achieving enduring health benefits.

The PF assessments in the exercise program included measures such as HGS, SLJ, a 4 × 10-meter speed/agility test, and the 20-meter shuttle run test, alongside accelerometer-based PA domains (LPA, MPA, VPA, MVPA, TPA, and SB). Average daily MVPA, assessed via GENEActiv accelerometers, was higher by 8.62 minutes in the exercise group than in the routine care group over the follow-up period. Although the routine care group showed improvement, the gamification group demonstrated notable progress, particularly in MVPA, which achieved the study’s primary objective despite the absence of significant between-group differences. Indeed, previous studies have shown that promoting high-intensity PA at an early age may have positive effects on body composition and PF levels in children over the long term, particularly in enhancing muscular strength [41]. Research on preschool PF by Lu et al [42] further emphasized that PA intensity plays a crucial role in fostering PF at this age. The benefits observed may be attributed to the specificity of lower resistance and endurance exercises used in the study (eg, body weight exercises rather than weightlifting), which likely contributed to improvements in muscular fitness as measured by the SLJ tests. Replacing LPA or SB time with MVPA appears to be an effective strategy for enhancing the muscular fitness of preschool-aged children [43].

The secondary outcomes, including anthropometric measures and body composition, did not exhibit significant changes; however, improvements in PF, as evidenced by gains in the SLJ and overall fitness (z-score), were noted. Additionally, enhancements in various PA metrics (LPA, MPA, TPA, and MVPA) highlighted the intervention’s effectiveness, with LPA and TPA showing statistically significant improvements. These findings align with longitudinal studies, such as those by Leppänen et al [43], that identified a strong association between higher levels of VPA/MVPA and improved CRF and muscular strength in young children. Similarly, our results mirror those of Fang et al’s [44], who reported positive correlations between MVPA and the SLJ test, as well as negative correlations between MVPA and the 4 × 10-meter speed/agility test. These findings underscore the critical role of PA in promoting fitness. Furthermore, Ha et al [45], in their study “Active 1+ FUN,” suggested that the “Active 1+ FUN” program was effective in improving the BMC of children. However, they concluded that further research is needed to explore how family-based initiatives could also effectively enhance PA behaviors.

Our results suggest that incorporating high-intensity PA at a young age offers lasting benefits, particularly in terms of CRF and muscular strength. These results are consistent with prior research highlighting the long-term positive effects of early PA on overall health [46,47]. However, sustaining PA over time requires the use of both intrinsic and extrinsic motivation strategies. Although gamification can effectively increase PA engagement, maintaining long-term adherence remains a significant challenge. Intrinsic motivation through enjoyment, a sense of mastery, and positive reinforcement can help children maintain an interest in PA as they age.

Gamification literature shows that engaging and interactive experiences have a stronger influence on PA than regular exercise methods among younger people who enjoy play and are at ease with digital technology [48]. Research on game-like interventions has found that children who take part in these activities show bigger gains in PA and PF than those following nongamified approaches [49]. By creating a playful yet structured environment, gamified programs increase motivation and encourage consistent engagement, which leads to better physical engagement [26]. For instance, Corepal et al [50] found that for some participants, gamification motivated adolescents to join in physically rewarding activities.

Our intervention distributed rewards like virtual accessories and sports-related items. This let children customize their avatars, which made them feel more connected to the program [49]. This reward system kept children coming back by setting up a clear path where they could open new “worlds” and move up levels to reach the “expert level.” Our program also involved families, which helped with factors that affect how well children stick with such programs. These include support from parents and children believing in themselves and having fun. All of these work together to create a good exercise experience that leads to lasting changes in behavior [43]. This shows how mixing game-like elements with family involvement is key. It meets the special motivational needs of young children, which in the end helps them stick with it and develop good health habits.

No previous research has looked at how gamification programs affect executive function outcomes, making it hard to compare results. We believe our study’s strong point is measuring the impact on executive function, which shows skills that help children focus, plan, prioritize, work toward goals, and control their behaviors and emotions, and serves as a better indicator of children’s mental health. What makes our findings even more convincing is that we looked at how many children in both the control and gamification groups had meaningful changes. We did not see big changes in the MOBAK KG battery [37]. However, the exercise group showed a clear trend in their favor. We think the groups did not differ much because children this age grow, develop, and mature.

One of the primary strengths of our study is the young age of the participants (mean age 5.0, SD 0.5 years), which allows for early intervention and potentially long-lasting impact on PA behaviors. Additionally, using objective PA measurements through accelerometers minimizes the subjective biases often encountered in questionnaire-based assessments. Accelerometers provide detailed insights into PA domains, capturing more precise data than self-reports. Another strength is the inclusion of the PREFIT fitness test [35], which is a validated and reliable measure of PF in this age group. This test enhances the robustness of our findings by ensuring that PF outcomes are accurately assessed in early childhood.

Our findings might be limited by the relatively small sample size (nonrepresentative), which may render some statistical analyses underpowered to detect significant differences. Additionally, some evaluators were not blinded to group allocation, which could introduce bias. The sample was drawn from a single primary care center in Pamplona, which may not be generalizable to the broader Spanish population due to potential regional variations in demographic characteristics and school environments. To improve representativeness, future studies should include multiple centers across diverse regions. Another limitation pertains to the use of accelerometers for measuring PA. Although accelerometers provide objective data, they may fail to capture some physical activities, such as cycling, swimming, or stair climbing, potentially leading to underestimation of overall PA levels. Additionally, the interpretation of PA intensity levels depends on predefined thresholds, which may vary by study and influence the consistency of results across different research contexts.

Parental involvement may have also influenced the outcomes, as young children required assistance from their parents to use the intervention platform. Variability in parental engagement likely impacted children’s adherence and motivation, contributing to variability in the results. To address these limitations, future research could incorporate objective tracking methods, such as wearable devices, to monitor PA. In this study, participants were required to complete at least 50% of the training sessions (12 of 24 sessions), with failure to meet this criterion resulting in the discontinuation of the study for that participant. Evidence from prior research supports a 50% adherence rate, which has been shown to improve PF, PA levels, and BMC. It is also important to note that preschoolers rely on parental supervision and may participate in other extracurricular activities. Tools like wearable devices could provide a more accurate representation of daily activities, reducing reliance on parental reporting. Furthermore, a multisite study design and the inclusion of diverse PA modalities could strengthen the generalizability of the findings.

### Conclusions

In summary, our study highlights that gamified family-based interventions have the potential to establish foundational PA behaviors in preschool children. By combining gamification with structured family support, these interventions can create a motivationally rich environment that encourages consistent engagement in PA. Incorporating such gamified strategies into school curricula and community programs may cultivate a culture of enjoyment around PA from an early age, helping to counter the natural tendency towards SB observed in later childhood stages. This approach could play a crucial role in the broader public health objectives of mitigating the prevalence of childhood obesity and promoting lifelong health.

## Supplementary material

10.2196/60185 Checklist 1 CONSORT-eHEALTH checklist (V 1.6.1).

## Abbreviations

ANCOVA: analysis of covariance
BMC: basic motor competencies
CDC-NHANES: Centers for Disease Control and Prevention’s National Health and Nutrition Examination Survey
CONSORT: Consolidated Standards of Reporting Trials
CRF: cardiorespiratory fitness
HGS: handgrip strength
LPA: light physical activity
MPA: moderate physical activity
MVPA: moderate to vigorous physical activity
OM: object movement
PA: physical activity
PF: physical fitness
SB: sedentary behavior
SLJ: standing long jump
SM: self-movement
TPA: total physical activity
VPA: vigorous physical activity
WC: waist circumference
WHO: World Health Organization
