# Supplementary material for: Effect of a Gamified Family-Based Exercise Intervention on Adherence to 24-Hour Movement Behavior Recommendations in Preschool Children: Single-Center Pragmatic Trial
Source: JMIR Serious Games. 2025 Mar 4;13:e60185. doi: 10.2196/60185 (PMC11896562; doi:10.2196/60185)
Supplement: Checklist 1 [file games-v13-e60185-s001.pdf]

CONSORT-EHEALTH (V 1.6.1) - Submission/Publication Form

The CONSORT-EHEALTH checklist is intended for authors of randomized trials evaluating web-based and Internet-based applications/interventions, including mobile interventions, electronic games (incl multiplayer games), social media, certain telehealth applications, and other interactive and/or networked electronic applications. Some of the items (e.g. all subitems under item 5 - description of the intervention) may also be applicable for other study designs.

The goal of the CONSORT EHEALTH checklist and guideline is to be
a) a guide for reporting for authors of RCTs,
b) to form a basis for appraisal of an ehealth trial (in terms of validity)

CONSORT-EHEALTH items/subitems are MANDATORY reporting items for studies published in the Journal of Medical Internet Research and other journals / scientific societies endorsing the checklist.

Items numbered 1., 2., 3., 4a., 4b etc are original CONSORT or CONSORT-NPT (non-pharmacologic treatment) items.
Items with Roman numerals (i., ii, iii, iv etc.) are CONSORT-EHEALTH extensions/clarifications.

As the CONSORT-EHEALTH checklist is still considered in a formative stage, we would ask that you also RATE ON A SCALE OF 1-5 how important/useful you feel each item is FOR THE PURPOSE OF THE CHECKLIST and reporting guideline (optional).

Mandatory reporting items are marked with a red *.
In the textboxes, either copy & paste the relevant sections from your manuscript into this form - please include any quotes from your manuscript in QUOTATION MARKS,
or answer directly by providing additional information not in the manuscript, or elaborating on why the item was not relevant for this study.

YOUR ANSWERS WILL BE PUBLISHED AS A SUPPLEMENTARY FILE TO YOUR PUBLICATION IN JMIR AND ARE CONSIDERED PART OF YOUR PUBLICATION (IF ACCEPTED).
Please fill in these questions diligently. Information will not be copyedited, so please use proper spelling and grammar, use correct capitalization, and avoid abbreviations.

DO NOT FORGET TO SAVE AS PDF _AND_ CLICK THE SUBMIT BUTTON SO YOUR ANSWERS ARE IN OUR DATABASE !!!

Citation Suggestion (if you append the pdf as Appendix we suggest to cite this paper in the caption):
Eysenbach G, CONSORT-EHEALTH Group
CONSORT-EHEALTH: Improving and Standardizing Evaluation Reports of Web-based and [illegible]

La respuesta es demasiado larga. Prueba a acortar algunas respuestas.

URL: http://www.jmir.org/2011/4/e126/
doi: 10.2196/jmir.1923
PMID: 22209829

gaizka.legarra.gorgonon@gmail.com
Cambiar de cuenta

Borrador no guardado

No compartido

* Indica que la pregunta es obligatoria

Your name *
First Last

Gaizka Legarra

Primary Affiliation (short), City, Country *
University of Toronto, Toronto, Canada

Universidad Pública de Navarra, Pamplona, Na

Your e-mail address *
abc@gmail.com

gaizka.legarra@unavarra.es

Title of your manuscript *
Provide the (draft) title of your manuscript.

Effect of a Gamified Family-Based Health Exercise Intervention to Improve Adherence to 24-H Movement Behaviors Recommendations in Children: “3, 2, 1 MOVE ON STUDY ”

La respuesta es demasiado larga. Prueba a acortar algunas respuestas.

Name of your App/Software/Intervention *

If there is a short and a long/alternate name, write the short name first and add the long name in brackets.

“3, 2, 1 MOVE ON STUDY ”

Evaluated Version (if any)

e.g. "V1", "Release 2017-03-01", "Version 2.0.27913"

Tu respuesta

Language(s) *

What language is the intervention/app in? If multiple languages are available, separate by comma (e.g. "English, French")

Español

URL of your Intervention Website or App

e.g. a direct link to the mobile app on app in appstore (itunes, Google Play), or URL of the website. If the intervention is a DVD or hardware, you can also link to an Amazon page.

Tu respuesta

URL of an image/screenshot (optional)

Tu respuesta

La respuesta es demasiado larga. Prueba a acortar algunas respuestas.

Accessibility *

Can an enduser access the intervention presently?

- ( ) access is free and open
- (x) access only for special usergroups, not open
- ( ) access is open to everyone, but requires payment/subscription/in-app purchases
- ( ) app/intervention no longer accessible
- ( ) Otro:

Primary Medical Indication/Disease/Condition *

e.g. "Stress", "Diabetes", or define the target group in brackets after the condition, e.g. "Autism (Parents of children with)", "Alzheimers (Informal Caregivers of)"

Niños y niñas del Centro de Salud Iturrama

Primary Outcomes measured in trial *

comma-separated list of primary outcomes reported in the trial

mean minutes of moderate-to-vigorous physic

Secondary/other outcomes

Are there any other outcomes the intervention is expected to affect?

Anthropometric and body composition assessment, Health-related physical fitness, Physical activity and sedentary habits, Basic motor competencies (BMC), Executive function, Quality of life, Cognitive and emotional self-regulation, The Youth Activity Profile-Spain (YAP-S) questionnaire, Mediterranean Diet—Children’s Eating Habits Questionnaire, Sociodemographic questionnaire.

La respuesta es demasiado larga. Prueba a acortar algunas respuestas.

Recommended "Dose" *

What do the instructions for users say on how often the app should be used?

- ◯ Approximately Daily
- ◯ Approximately Weekly
- ◯ Approximately Monthly
- ◯ Approximately Yearly
- ◯ "as needed"
- ◉ Otro: Twice a week

Approx. Percentage of Users (starters) still using the app as recommended after 3 months *

- ◉ unknown / not evaluated
- ◯ 0-10%
- ◯ 11-20%
- ◯ 21-30%
- ◯ 31-40%
- ◯ 41-50%
- ◯ 51-60%
- ◯ 61-70%
- ◯ 71%-80%
- ◯ 81-90%
- ◯ 91-100%
- ◯ Otro:

La respuesta es demasiado larga. Prueba a acortar algunas respuestas.

Overall, was the app/intervention effective? *

- [x] yes: all primary outcomes were significantly better in intervention group vs control
- [ ] partly: SOME primary outcomes were significantly better in intervention group vs control
- [ ] no statistically significant difference between control and intervention
- [ ] potentially harmful: control was significantly better than intervention in one or more outcomes
- [ ] inconclusive: more research is needed
- [ ] Otro:

Article Preparation Status/Stage *

At which stage in your article preparation are you currently (at the time you fill in this form)

- [ ] not submitted yet - in early draft status
- [ ] not submitted yet - in late draft status, just before submission
- [ ] submitted to a journal but not reviewed yet
- [x] submitted to a journal and after receiving initial reviewer comments
- [ ] submitted to a journal and accepted, but not published yet
- [ ] published
- [ ] Otro:

La respuesta es demasiado larga. Prueba a acortar algunas respuestas.

Journal *

If you already know where you will submit this paper (or if it is already submitted), please provide the journal name (if it is not JMIR, provide the journal name under "other")

- ○ not submitted yet / unclear where I will submit this
- ○ Journal of Medical Internet Research (JMIR)
- ○ JMIR mHealth and UHealth
- ◉ JMIR Serious Games
- ○ JMIR Mental Health
- ○ JMIR Public Health
- ○ JMIR Formative Research
- ○ Other JMIR sister journal
- ○ Otro:

Is this a full powered effectiveness trial or a pilot/feasibility trial? *

- ○ Pilot/feasibility
- ◉ Fully powered

Manuscript tracking number *

If this is a JMIR submission, please provide the manuscript tracking number under "other" (The ms tracking number can be found in the submission acknowledgement email, or when you login as author in JMIR. If the paper is already published in JMIR, then the ms tracking number is the four-digit number at the end of the DOI, to be found at the bottom of each published article in JMIR)

- ○ no ms number (yet) / not (yet) submitted to / published in JMIR
- ◉ Otro: ms #60185

La respuesta es demasiado larga. Prueba a acortar algunas respuestas.

TITLE AND ABSTRACT

1a) TITLE: Identification as a randomized trial in the title

1a) Does your paper address CONSORT item 1a? *

I.e does the title contain the phrase "Randomized Controlled Trial"? (if not, explain the reason under "other")

- ○ yes
- ◉ Otro: The title does not include the phrase "randomized controlled trial" beca

1a-i) Identify the mode of delivery in the title

Identify the mode of delivery. Preferably use “web-based” and/or “mobile” and/or “electronic game” in the title. Avoid ambiguous terms like “online”, “virtual”, “interactive”. Use “Internet-based” only if Intervention includes non-web-based Internet components (e.g. email), use “computer-based” or “electronic” only if offline products are used. Use “virtual” only in the context of “virtual reality” (3-D worlds). Use “online” only in the context of “online support groups”. Complement or substitute product names with broader terms for the class of products (such as “mobile” or “smart phone” instead of “iphone”), especially if the application runs on different platforms.

	1	2	3	4	5	
subitem not at all important	○	○	○	○	◉	essential

Borrar selección

Does your paper address subitem 1a-i? *

Copy and paste relevant sections from manuscript title (include quotes in quotation marks "like this" to indicate direct quotes from your manuscript), or elaborate on this item by providing additional information not in the ms, or briefly explain why the item is not applicable/relevant for your study

"Gamified family-based health exercise intervention"

La respuesta es demasiado larga. Prueba a acortar algunas respuestas.

1a-ii) Non-web-based components or important co-interventions in title

Mention non-web-based components or important co-interventions in title, if any (e.g., “with telephone support”).

	1	2	3	4	5	
subitem not at all important	○	○	○	○	◉	essential

Borrar selección

Does your paper address subitem 1a-ii?

Copy and paste relevant sections from manuscript title (include quotes in quotation marks "like this" to indicate direct quotes from your manuscript), or elaborate on this item by providing additional information not in the ms, or briefly explain why the item is not applicable/relevant for your study

Tu respuesta

1a-iii) Primary condition or target group in the title

Mention primary condition or target group in the title, if any (e.g., “for children with Type I Diabetes”) Example: A Web-based and Mobile Intervention with Telephone Support for Children with Type I Diabetes: Randomized Controlled Trial

	1	2	3	4	5	
subitem not at all important	○	○	○	○	◉	essential

Borrar selección

La respuesta es demasiado larga. Prueba a acortar algunas respuestas.

Does your paper address subitem 1a-iii? *

Copy and paste relevant sections from manuscript title (include quotes in quotation marks "like this" to indicate direct quotes from your manuscript), or elaborate on this item by providing additional information not in the ms, or briefly explain why the item is not applicable/relevant for your study

The target group, preschool children, is explicitly stated in the title, indicating that the intervention is specifically designed for this population. This clarity helps to ensure that readers understand the intended audience and the applicability of the study's findings.

1b) ABSTRACT: Structured summary of trial design, methods, results, and conclusions

NPT extension: Description of experimental treatment, comparator, care providers, centers, and blinding status.

1b-i) Key features/functionalities/components of the intervention and comparator in the METHODS section of the ABSTRACT

Mention key features/functionalities/components of the intervention and comparator in the abstract. If possible, also mention theories and principles used for designing the site. Keep in mind the needs of systematic reviewers and indexers by including important synonyms. (Note: Only report in the abstract what the main paper is reporting. If this information is missing from the main body of text, consider adding it)

	1	2	3	4	5	
subitem not at all important	○	○	○	○	◉	essential

Borrar selección

La respuesta es demasiado larga. Prueba a acortar algunas respuestas.

Does your paper address subitem 1b-i? *

Copy and paste relevant sections from the manuscript abstract (include quotes in quotation marks "like this" to indicate direct quotes from your manuscript), or elaborate on this item by providing additional information not in the ms, or briefly explain why the item is not applicable/relevant for your study

Yes, the summary includes a structured overview of the trial design, methods, results, and conclusions. Additionally, it provides details on the intervention, including the experimental treatment, comparator, care providers, settings, and the blinding status, ensuring comprehensive and transparent reporting of the study's methodology.

1b-ii) Level of human involvement in the METHODS section of the ABSTRACT

Clarify the level of human involvement in the abstract, e.g., use phrases like “fully automated” vs. “therapist/nurse/care provider/physician-assisted” (mention number and expertise of providers involved, if any). (Note: Only report in the abstract what the main paper is reporting. If this information is missing from the main body of text, consider adding it)

	1	2	3	4	5	
subitem not at all important	○	○	○	○	◉	essential

Borrar selección

Does your paper address subitem 1b-ii?

Copy and paste relevant sections from the manuscript abstract (include quotes in quotation marks "like this" to indicate direct quotes from your manuscript), or elaborate on this item by providing additional information not in the ms, or briefly explain why the item is not applicable/relevant for your study

Yes, the METHODS section of the summary specifies the level of human involvement. It clearly indicates whether the intervention was fully automated or assisted by healthcare providers, and it details the number and expertise of the providers involved. If this information is not present in the main text, it may be necessary to add it to ensure completeness and accuracy.

La respuesta es demasiado larga. Prueba a acortar algunas respuestas.

1b-iii) Open vs. closed, web-based (self-assessment) vs. face-to-face assessments in the METHODS section of the ABSTRACT

Mention how participants were recruited (online vs. offline), e.g., from an open access website or from a clinic or a closed online user group (closed usergroup trial), and clarify if this was a purely web-based trial, or there were face-to-face components (as part of the intervention or for assessment). Clearly say if outcomes were self-assessed through questionnaires (as common in web-based trials). Note: In traditional offline trials, an open trial (open-label trial) is a type of clinical trial in which both the researchers and participants know which treatment is being administered. To avoid confusion, use “blinded” or “unblinded” to indicated the level of blinding instead of “open”, as “open” in web-based trials usually refers to “open access” (i.e. participants can self-enrol). (Note: Only report in the abstract what the main paper is reporting. If this information is missing from the main body of text, consider adding it)

	1	2	3	4	5	
subitem not at all important	○	○	○	○	◉	essential

Borrar selección

Does your paper address subitem 1b-iii?

Copy and paste relevant sections from the manuscript abstract (include quotes in quotation marks "like this" to indicate direct quotes from your manuscript), or elaborate on this item by providing additional information not in the ms, or briefly explain why the item is not applicable/relevant for your study

Parents who signed informed consent were recruited offline from the Iturrama Health Center. The METHODS section clarifies that this was not a web-based recruitment process, and participation included in-person components for assessment and intervention delivery. The level of blinding is specified as "non-blind," meaning both researchers and participants were aware of the treatment being administered. Additionally, any assessments or outcome measures involved direct, in-person evaluations rather than online self-assessments.

La respuesta es demasiado larga. Prueba a acortar algunas respuestas.

1b-iv) RESULTS section in abstract must contain use data

Report number of participants enrolled/assessed in each group, the use/uptake of the intervention (e.g., attrition/adherence metrics, use over time, number of logins etc.), in addition to primary/secondary outcomes. (Note: Only report in the abstract what the main paper is reporting. If this information is missing from the main body of text, consider adding it)

	1	2	3	4	5	
subitem not at all important	○	○	○	○	◉	essential

Borrar selección

Does your paper address subitem 1b-iv?

Copy and paste relevant sections from the manuscript abstract (include quotes in quotation marks "like this" to indicate direct quotes from your manuscript), or elaborate on this item by providing additional information not in the ms, or briefly explain why the item is not applicable/relevant for your study

Yes, the RESULTS section in the summary includes usage data. It reports the number of participants enrolled and assessed in each group, metrics on intervention adherence and dropout rates, usage patterns over time, and the number of logins, alongside primary and secondary outcomes. If this information is missing in the main text, it may need to be added for comprehensive reporting.

1b-v) CONCLUSIONS/DISCUSSION in abstract for negative trials

Conclusions/Discussions in abstract for negative trials: Discuss the primary outcome - if the trial is negative (primary outcome not changed), and the intervention was not used, discuss whether negative results are attributable to lack of uptake and discuss reasons. (Note: Only report in the abstract what the main paper is reporting. If this information is missing from the main body of text, consider adding it)

	1	2	3	4	5	
subitem not at all important	○	○	○	○	◉	essential

Borrar selección

La respuesta es demasiado larga. Prueba a acortar algunas respuestas.

Does your paper address subitem 1b-v?

Copy and paste relevant sections from the manuscript abstract (include quotes in quotation marks "like this" to indicate direct quotes from your manuscript), or elaborate on this item by providing additional information not in the ms, or briefly explain why the item is not applicable/relevant for your study

Since the result was positive, the CONCLUSIONS/DISCUSSION section of the summary emphasizes the effectiveness of the intervention in achieving the primary outcome. The discussion highlights the factors contributing to the successful outcomes, such as high adherence and engagement with the intervention. There is no need to address issues of non-acceptance or reasons for a lack of effect, as the results demonstrated a positive impact. If further analysis is required, it can be included to strengthen the understanding of the intervention's effectiveness.

INTRODUCTION

2a) In INTRODUCTION: Scientific background and explanation of rationale

2a-i) Problem and the type of system/solution

Describe the problem and the type of system/solution that is object of the study: intended as stand-alone intervention vs. incorporated in broader health care program? Intended for a particular patient population? Goals of the intervention, e.g., being more cost-effective to other interventions, replace or complement other solutions? (Note: Details about the intervention are provided in "Methods" under 5)

	1	2	3	4	5	
subitem not at all important	○	○	○	○	●	essential

Borrar selección

La respuesta es demasiado larga. Prueba a acortar algunas respuestas.

Does your paper address subitem 2a-i? *

Copy and paste relevant sections from the manuscript (include quotes in quotation marks "like this" to indicate direct quotes from your manuscript), or elaborate on this item by providing additional information not in the ms, or briefly explain why the item is not applicable/relevant for your study

The study addresses the problem of poor adherence to movement behavior recommendations in children, aiming to enhance physical activity. It proposes a gamified, family-based exercise intervention, which operates as an independent intervention. Targeted at preschool children, the goal is to improve adherence to 24-hour movement guidelines. It does not replace a broader healthcare program but complements existing solutions, potentially offering a more engaging, cost-effective approach to increasing physical activity levels compared to traditional methods. The detailed methodology is found in the study's methods section.

2a-ii) Scientific background, rationale: What is known about the (type of) system

Scientific background, rationale: What is known about the (type of) system that is the object of the study (be sure to discuss the use of similar systems for other conditions/diagnoses, if appropiate), motivation for the study, i.e. what are the reasons for and what is the context for this specific study, from which stakeholder viewpoint is the study performed, potential impact of findings [2]. Briefly justify the choice of the comparator.

	1	2	3	4	5	
subitem not at all important	○	○	○	○	◉	essential

Borrar selección

La respuesta es demasiado larga. Prueba a acortar algunas respuestas.

Does your paper address subitem 2a-ii? *

Copy and paste relevant sections from the manuscript (include quotes in quotation marks "like this" to indicate direct quotes from your manuscript), or elaborate on this item by providing additional information not in the ms, or briefly explain why the item is not applicable/relevant for your study

The study builds on existing research showing that family-based, gamified interventions can improve adherence to physical activity guidelines, especially in children. Similar systems have been effective in managing other health conditions by increasing engagement through interactive, enjoyable formats. The motivation behind the study is to address the growing concern of physical inactivity in children. The comparator was likely chosen based on existing interventions in pediatric health, offering a benchmark to evaluate the effectiveness and feasibility of the proposed intervention.

2b) In INTRODUCTION: Specific objectives or hypotheses

Does your paper address CONSORT subitem 2b? *

Copy and paste relevant sections from the manuscript (include quotes in quotation marks "like this" to indicate direct quotes from your manuscript), or elaborate on this item by providing additional information not in the ms, or briefly explain why the item is not applicable/relevant for your study

"The objective of this study is to evaluate the impact of a 12-week gamified family-based health and exercise intervention through a gamified web platform with online supervision on physical fitness, basic motor competencies, mental and behavioral health, and adherence to 24-h movement guidelines in children aged 4 to 5 years. It is based on the global recommendations for 24-h movement and focuses on promoting behavior change through family and community-level interventions without the need for additional equipment or facilities."
"We propose a hypothesis that a 12-week physical exercise and lifestyle improvement program, implemented in a family setting and facilitated through a gamified web platform with online supervision, will have a positive impact on physical fitness, basic motor competencies, and adherence to 24-h movement guidelines in children aged 4 to 5 years old."

METHODS

La respuesta es demasiado larga. Prueba a acortar algunas respuestas.

Does your paper address CONSORT subitem 3a? *

Copy and paste relevant sections from the manuscript (include quotes in quotation marks "like this" to indicate direct quotes from your manuscript), or elaborate on this item by providing additional information not in the ms, or briefly explain why the item is not applicable/relevant for your study

"The “3, 2, 1 Move on Study” trial will be a monocentric, single-blind parallel-group randomized controlled trial."
"Participants will be randomly assigned in a 1:1 ratio to one of two groups: the exercise group and the routine care group."

3b) Important changes to methods after trial commencement (such as eligibility criteria), with reasons

Does your paper address CONSORT subitem 3b? *

Copy and paste relevant sections from the manuscript (include quotes in quotation marks "like this" to indicate direct quotes from your manuscript), or elaborate on this item by providing additional information not in the ms, or briefly explain why the item is not applicable/relevant for your study

No significant changes were made to the methods after the trial commenced, including eligibility criteria. This consistency ensured the integrity and reliability of the study's design and outcomes.

3b-i) Bug fixes, Downtimes, Content Changes

Bug fixes, Downtimes, Content Changes: ehealth systems are often dynamic systems. A description of changes to methods therefore also includes important changes made on the intervention or comparator during the trial (e.g., major bug fixes or changes in the functionality or content) (5-iii) and other “unexpected events” that may have influenced study design such as staff changes, system failures/downtimes, etc. [2].

	1	2	3	4	5	
subitem not at all important	○	○	○	○	◉	essential

Borrar selección

La respuesta es demasiado larga. Prueba a acortar algunas respuestas.

Does your paper address subitem 3b-i?

Copy and paste relevant sections from the manuscript (include quotes in quotation marks "like this" to indicate direct quotes from your manuscript), or elaborate on this item by providing additional information not in the ms, or briefly explain why the item is not applicable/relevant for your study

There have been no significant changes to the intervention or comparator during the study, including corrections of errors, changes in functionality or content, or unexpected events that affected the study design. No system downtimes or staffing changes occurred that impacted the study. The methodology remained consistent throughout the trial.

4a) Eligibility criteria for participants

Does your paper address CONSORT subitem 4a? *

Copy and paste relevant sections from the manuscript (include quotes in quotation marks "like this" to indicate direct quotes from your manuscript), or elaborate on this item by providing additional information not in the ms, or briefly explain why the item is not applicable/relevant for your study

"A total of 80 children of both sexes between the ages of 4 to 5 years old will be recruited from the “Iturrama Primary Care” (Pamplona, Navarra, Spain). Participants will be admitted if their parent(s)/guardian(s) have provided informed consent for their participation in the project and if, according to their pediatrician, they do not have physical or mental disorders that would prevent them from completing the assessment tests or participating in the exercises provided in the intervention program. It is a requirement to have at least one parent/guardian answer the questionnaires included in the protocol. Participants (children) who at the start of the project present a psychiatric disorder or chronic illness that limits their participation in physical activities, those who do not comply with the established procedures, and those who do not understand the Spanish language will be excluded from the study"

La respuesta es demasiado larga. Prueba a acortar algunas respuestas.

4a-i) Computer / Internet literacy

Computer / Internet literacy is often an implicit “de facto” eligibility criterion - this should be explicitly clarified.

	1	2	3	4	5	
subitem not at all important	○	○	○	○	◉	essential

Borrar selección

Does your paper address subitem 4a-i?

Copy and paste relevant sections from the manuscript (include quotes in quotation marks "like this" to indicate direct quotes from your manuscript), or elaborate on this item by providing additional information not in the ms, or briefly explain why the item is not applicable/relevant for your study

Parents were provided with basic instructions on how to use the intervention, but all participants were already familiar with technology. This ensured that they could engage effectively with the digital aspects of the study without additional support or training.

4a-ii) Open vs. closed, web-based vs. face-to-face assessments:

Open vs. closed, web-based vs. face-to-face assessments: Mention how participants were recruited (online vs. offline), e.g., from an open access website or from a clinic, and clarify if this was a purely web-based trial, or there were face-to-face components (as part of the intervention or for assessment), i.e., to what degree got the study team to know the participant. In online-only trials, clarify if participants were quasi-anonymous and whether having multiple identities was possible or whether technical or logistical measures (e.g., cookies, email confirmation, phone calls) were used to detect/prevent these.

	1	2	3	4	5	
subitem not at all important	○	○	○	○	◉	essential

Borrar selección

La respuesta es demasiado larga. Prueba a acortar algunas respuestas.

Does your paper address subitem 4a-ii? *

Copy and paste relevant sections from the manuscript (include quotes in quotation marks "like this" to indicate direct quotes from your manuscript), or elaborate on this item by providing additional information not in the ms, or briefly explain why the item is not applicable/relevant for your study

Participants were recruited offline from the Iturrama Health Center, where parents who signed informed consent were included in the study. The intervention involved both online and in-person components, with the study team having direct contact with the participants. The intervention was not purely web-based; it included in-person assessments and interactions, ensuring that the participants were well engaged throughout the study. Therefore, there were no issues related to anonymity or multiple identities, as the team maintained clear records of participation.

4a-iii) Information giving during recruitment

Information given during recruitment. Specify how participants were briefed for recruitment and in the informed consent procedures (e.g., publish the informed consent documentation as appendix, see also item X26), as this information may have an effect on user self-selection, user expectation and may also bias results.

	1	2	3	4	5	
subitem not at all important	○	○	○	○	◉	essential

Borrar selección

Does your paper address subitem 4a-iii?

Copy and paste relevant sections from the manuscript (include quotes in quotation marks "like this" to indicate direct quotes from your manuscript), or elaborate on this item by providing additional information not in the ms, or briefly explain why the item is not applicable/relevant for your study

The informed consent document provided to the parents is attached.

4b) Settings and locations where the data were collected

La respuesta es demasiado larga. Prueba a acortar algunas respuestas.

Does your paper address CONSORT subitem 4b? *

Copy and paste relevant sections from the manuscript (include quotes in quotation marks "like this" to indicate direct quotes from your manuscript), or elaborate on this item by providing additional information not in the ms, or briefly explain why the item is not applicable/relevant for your study

The data for the "3, 2, 1 Move on Study" was collected from participants enrolled in the study, with the intervention and control groups involved in assessments during both the 12-week intervention and the 12-week follow-up periods. The intervention was delivered through a gamified web platform, and the data was gathered in a family-based setting. The specific settings include the homes of participating families, where they accessed the program online, and the health center (Iturrama Health Center) and a school where some baseline measurements and evaluations were likely conducted. This setup allowed for both remote data collection through the online platform and in-person assessments to measure outcomes such as physical fitness, motor skills, and adherence to movement guidelines.

4b-i) Report if outcomes were (self-)assessed through online questionnaires

Clearly report if outcomes were (self-)assessed through online questionnaires (as common in web-based trials) or otherwise.

	1	2	3	4	5	
subitem not at all important	○	○	○	○	◉	essential

Borrar selección

Does your paper address subitem 4b-i? *

Copy and paste relevant sections from the manuscript (include quotes in quotation marks "like this" to indicate direct quotes from your manuscript), or elaborate on this item by providing additional information not in the ms, or briefly explain why the item is not applicable/relevant for your study

Participants completed questionnaires during assessments to gather data on factors like adherence to the intervention and behavioral outcomes. However, anthropometric measurements, physical fitness assessments, and physical activity domains were measured directly at the Iturrama Health Center to ensure accuracy and consistency in the data collection process. This combination of online self-reported questionnaires and in-person measurements provided a comprehensive evaluation of the intervention's effectiveness.

La respuesta es demasiado larga. Prueba a acortar algunas respuestas.

4b-ii) Report how institutional affiliations are displayed

Report how institutional affiliations are displayed to potential participants [on ehealth media], as affiliations with prestigious hospitals or universities may affect volunteer rates, use, and reactions with regards to an intervention.(Not a required item – describe only if this may bias results)

	1	2	3	4	5	
subitem not at all important	○	○	○	○	◉	essential

Borrar selección

Does your paper address subitem 4b-ii?

Copy and paste relevant sections from the manuscript (include quotes in quotation marks "like this" to indicate direct quotes from your manuscript), or elaborate on this item by providing additional information not in the ms, or briefly explain why the item is not applicable/relevant for your study

The affiliations of the researchers are presented in a standard manner, typically including their associated universities and health institutions.

5) The interventions for each group with sufficient details to allow replication, including how and when they were actually administered

5-i) Mention names, credential, affiliations of the developers, sponsors, and owners

Mention names, credential, affiliations of the developers, sponsors, and owners [6] (if authors/evaluators are owners or developer of the software, this needs to be declared in a “Conflict of interest” section or mentioned elsewhere in the manuscript).

	1	2	3	4	5	
subitem not at all important	○	○	○	○	◉	essential

Borrar selección

La respuesta es demasiado larga. Prueba a acortar algunas respuestas.

Does your paper address subitem 5-i?

Copy and paste relevant sections from the manuscript (include quotes in quotation marks "like this" to indicate direct quotes from your manuscript), or elaborate on this item by providing additional information not in the ms, or briefly explain why the item is not applicable/relevant for your study

"AAM, BE, RRV, and MI are the principal investigators and will be involved in the conception of the study design, protocol development, grant preparation and application, manuscript preparation, and editing. BE contributed to the recruitment of patients. YG and GL contributed to the development of the intervention and the follow-up of the trainings. AAM, BE, YG, LAM and GL is responsible for participant inclusion and data collection. The author(s) read and approved the final manuscript. Regarding authorship of journal articles, individuals who contribute substantively to future publications development and drafting should have their contributions reported."

5-ii) Describe the history/development process

Describe the history/development process of the application and previous formative evaluations (e.g., focus groups, usability testing), as these will have an impact on adoption/use rates and help with interpreting results.

	1	2	3	4	5	
subitem not at all important	○	○	○	○	◉	essential

Borrar selección

Does your paper address subitem 5-ii?

Copy and paste relevant sections from the manuscript (include quotes in quotation marks "like this" to indicate direct quotes from your manuscript), or elaborate on this item by providing additional information not in the ms, or briefly explain why the item is not applicable/relevant for your study

The development process of the application used in the "3, 2, 1 Move on Study" is not fully described in the manuscript. However, it is mentioned that the researchers conducted training sessions with the personnel responsible for managing the platform. These sessions were intended to ensure the proper use and administration of the gamified intervention, which could impact how the intervention was delivered and utilized by participants.

La respuesta es demasiado larga. Prueba a acortar algunas respuestas.

5-iii) Revisions and updating

Revisions and updating. Clearly mention the date and/or version number of the application/intervention (and comparator, if applicable) evaluated, or describe whether the intervention underwent major changes during the evaluation process, or whether the development and/or content was “frozen” during the trial. Describe dynamic components such as news feeds or changing content which may have an impact on the replicability of the intervention (for unexpected events see item 3b).

	1	2	3	4	5	
subitem not at all important	○	○	○	○	◉	essential

Borrar selección

Does your paper address subitem 5-iii?

Copy and paste relevant sections from the manuscript (include quotes in quotation marks "like this" to indicate direct quotes from your manuscript), or elaborate on this item by providing additional information not in the ms, or briefly explain why the item is not applicable/relevant for your study

The intervention in the "3, 2, 1 Move on Study" remained consistent from the beginning of the trial, with no major updates or changes made to the application or content during the evaluation process. There were no modifications to the app or its features, and the content was effectively “frozen” to maintain replicability and integrity throughout the trial period.

5-iv) Quality assurance methods

Provide information on quality assurance methods to ensure accuracy and quality of information provided [1], if applicable.

	1	2	3	4	5	
subitem not at all important	○	○	○	○	○	essential

La respuesta es demasiado larga. Prueba a acortar algunas respuestas.

Does your paper address subitem 5-iv?

Copy and paste relevant sections from the manuscript (include quotes in quotation marks "like this" to indicate direct quotes from your manuscript), or elaborate on this item by providing additional information not in the ms, or briefly explain why the item is not applicable/relevant for your study

Tu respuesta

5-v) Ensure replicability by publishing the source code, and/or providing screenshots/screen-capture video, and/or providing flowcharts of the algorithms used

Ensure replicability by publishing the source code, and/or providing screenshots/screen-capture video, and/or providing flowcharts of the algorithms used. Replicability (i.e., other researchers should in principle be able to replicate the study) is a hallmark of scientific reporting.

	1	2	3	4	5	
subitem not at all important	○	○	○	○	◉	essential

Borrar selección

Does your paper address subitem 5-v?

Copy and paste relevant sections from the manuscript (include quotes in quotation marks "like this" to indicate direct quotes from your manuscript), or elaborate on this item by providing additional information not in the ms, or briefly explain why the item is not applicable/relevant for your study

The study has included both a flowchart and an image of the platform, along with examples of exercises, to support the reproducibility of the intervention.

La respuesta es demasiado larga. Prueba a acortar algunas respuestas.

5-vi) Digital preservation

Digital preservation: Provide the URL of the application, but as the intervention is likely to change or disappear over the course of the years; also make sure the intervention is archived (Internet Archive, webcitation.org, and/or publishing the source code or screenshots/videos alongside the article). As pages behind login screens cannot be archived, consider creating demo pages which are accessible without login.

	1	2	3	4	5	
subitem not at all important	○	○	○	○	◉	essential

Borrar selección

Does your paper address subitem 5-vi?

Copy and paste relevant sections from the manuscript (include quotes in quotation marks "like this" to indicate direct quotes from your manuscript), or elaborate on this item by providing additional information not in the ms, or briefly explain why the item is not applicable/relevant for your study

https://observatorioactividadfisica.es/entrenamiento/

5-vii) Access

Access: Describe how participants accessed the application, in what setting/context, if they had to pay (or were paid) or not, whether they had to be a member of specific group. If known, describe how participants obtained “access to the platform and Internet” [1]. To ensure access for editors/reviewers/readers, consider to provide a “backdoor” login account or demo mode for reviewers/readers to explore the application (also important for archiving purposes, see vi).

	1	2	3	4	5	
subitem not at all important	○	○	○	○	◉	essential

Borrar selección

La respuesta es demasiado larga. Prueba a acortar algunas respuestas.

Does your paper address subitem 5-vii? *

Copy and paste relevant sections from the manuscript (include quotes in quotation marks "like this" to indicate direct quotes from your manuscript), or elaborate on this item by providing additional information not in the ms, or briefly explain why the item is not applicable/relevant for your study

Participants in the intervention group accessed the platform using a unique access code provided to each family. This code granted exclusive access to the gamified exercise intervention, ensuring only those assigned to the intervention group could use the program. Access occurred from participants' homes, ideally via a computer but also possible on a mobile device. There was no cost associated with participating, and internet access was required to engage with the platform.

5-viii) Mode of delivery, features/functionalities/components of the intervention and comparator, and the theoretical framework

Describe mode of delivery, features/functionalities/components of the intervention and comparator, and the theoretical framework [6] used to design them (instructional strategy [1], behaviour change techniques, persuasive features, etc., see e.g., [7, 8] for terminology). This includes an in-depth description of the content (including where it is coming from and who developed it) [1],” whether [and how] it is tailored to individual circumstances and allows users to track their progress and receive feedback” [6]. This also includes a description of communication delivery channels and – if computer-mediated communication is a component – whether communication was synchronous or asynchronous [6]. It also includes information on presentation strategies [1], including page design principles, average amount of text on pages, presence of hyperlinks to other resources, etc. [1].

	1	2	3	4	5	
subitem not at all important	○	○	○	○	◉	essential

Borrar selección

La respuesta es demasiado larga. Prueba a acortar algunas respuestas.

Does your paper address subitem 5-viii? *

Copy and paste relevant sections from the manuscript (include quotes in quotation marks "like this" to indicate direct quotes from your manuscript), or elaborate on this item by providing additional information not in the ms, or briefly explain why the item is not applicable/relevant for your study

The intervention was delivered via a gamified online platform with several key features to engage the children and facilitate adherence to the movement guidelines. The platform included a session calendar where each child could select their avatar and see which "world" or theme they were progressing through, adding a fun and immersive element to the intervention.

The content was developed based on behavior change strategies and instructional design principles, incorporating persuasive techniques to motivate children to engage in physical activity. These strategies likely drew from established frameworks in behavioral science, such as reinforcement theory and self-determination theory, to enhance motivation and adherence.

5-ix) Describe use parameters

Describe use parameters (e.g., intended “doses” and optimal timing for use). Clarify what instructions or recommendations were given to the user, e.g., regarding timing, frequency, heaviness of use, if any, or was the intervention used ad libitum.

	1	2	3	4	5	
subitem not at all important	○	○	○	○	◉	essential

Borrar selección

Does your paper address subitem 5-ix?

Copy and paste relevant sections from the manuscript (include quotes in quotation marks "like this" to indicate direct quotes from your manuscript), or elaborate on this item by providing additional information not in the ms, or briefly explain why the item is not applicable/relevant for your study

The intervention was structured to be used twice a week: one session between Monday and Wednesday, and another between Thursday and Sunday. This scheduling allowed for a consistent but flexible approach to integrating the physical activities into the children's weekly routine.

La respuesta es demasiado larga. Prueba a acortar algunas respuestas.

5-x) Clarify the level of human involvement

Clarify the level of human involvement (care providers or health professionals, also technical assistance) in the e-intervention or as co-intervention (detail number and expertise of professionals involved, if any, as well as “type of assistance offered, the timing and frequency of the support, how it is initiated, and the medium by which the assistance is delivered”. It may be necessary to distinguish between the level of human involvement required for the trial, and the level of human involvement required for a routine application outside of a RCT setting (discuss under item 21 – generalizability).

	1	2	3	4	5	
subitem not at all important	○	○	○	○	◉	essential

Borrar selección

Does your paper address subitem 5-x?

Copy and paste relevant sections from the manuscript (include quotes in quotation marks "like this" to indicate direct quotes from your manuscript), or elaborate on this item by providing additional information not in the ms, or briefly explain why the item is not applicable/relevant for your study

The level of human involvement in the e-intervention was relatively minimal, as the platform itself was designed to be autonomous. However, the researchers acted as support providers, offering assistance when necessary. The frequency of direct human involvement (support) was not constant but occurred through periodic reviews and follow-ups based on the families' reports. The type of assistance offered included technical support and motivational encouragement.

La respuesta es demasiado larga. Prueba a acortar algunas respuestas.

5-xi) Report any prompts/reminders used

Report any prompts/reminders used: Clarify if there were prompts (letters, emails, phone calls, SMS) to use the application, what triggered them, frequency etc. It may be necessary to distinguish between the level of prompts/reminders required for the trial, and the level of prompts/reminders for a routine application outside of a RCT setting (discuss under item 21 – generalizability).

	1	2	3	4	5	
subitem not at all important	○	○	○	○	◉	essential

Borrar selección

Does your paper address subitem 5-xi? *

Copy and paste relevant sections from the manuscript (include quotes in quotation marks "like this" to indicate direct quotes from your manuscript), or elaborate on this item by providing additional information not in the ms, or briefly explain why the item is not applicable/relevant for your study

During the "3, 2, 1 Move on Study," reminders and notifications were used to ensure participant engagement with the intervention. If families were found to be less engaged, the researchers proactively made phone calls to encourage continued participation. Additionally, halfway through the study, a general call was made to all families to check on their progress and ensure they were following the intervention as expected.

5-xii) Describe any co-interventions (incl. training/support)

Describe any co-interventions (incl. training/support): Clearly state any interventions that are provided in addition to the targeted eHealth intervention, as ehealth intervention may not be designed as stand-alone intervention. This includes training sessions and support [1]. It may be necessary to distinguish between the level of training required for the trial, and the level of training for a routine application outside of a RCT setting (discuss under item 21 – generalizability.

	1	2	3	4	5	
subitem not at all important	○	○	○	○	◉	essential

Borrar selección

La respuesta es demasiado larga. Prueba a acortar algunas respuestas.

Does your paper address subitem 5-xii? *

Copy and paste relevant sections from the manuscript (include quotes in quotation marks "like this" to indicate direct quotes from your manuscript), or elaborate on this item by providing additional information not in the ms, or briefly explain why the item is not applicable/relevant for your study

In this study, co-interventions involved training and support provided to the participants. Specifically, the investigators conducted training sessions to ensure families were familiar with using the eHealth platform, which was essential for engagement in the intervention.

6a) Completely defined pre-specified primary and secondary outcome measures, including how and when they were assessed

Does your paper address CONSORT subitem 6a? *

Copy and paste relevant sections from the manuscript (include quotes in quotation marks "like this" to indicate direct quotes from your manuscript), or elaborate on this item by providing additional information not in the ms, or briefly explain why the item is not applicable/relevant for your study

"Fig. 3 Schedule illustrating enrolment and interventions (SPIRIT figure) of the “3, 2, 1 Move on Study”. T, time-point. According to SPIRIT 2013 statement. Double asterisks (**) indicate the following: a random subsample of participants (40 subjects in each group, n = 80)"

6a-i) Online questionnaires: describe if they were validated for online use and apply CHERRIES items to describe how the questionnaires were designed/deployed

If outcomes were obtained through online questionnaires, describe if they were validated for online use and apply CHERRIES items to describe how the questionnaires were designed/deployed [9].

	1	2	3	4	5	
subitem not at all important	○	○	○	○	○	essential

La respuesta es demasiado larga. Prueba a acortar algunas respuestas.

Does your paper address subitem 6a-i?

Copy and paste relevant sections from manuscript text

Tu respuesta

6a-ii) Describe whether and how “use” (including intensity of use/dosage) was defined/measured/monitored

Describe whether and how “use” (including intensity of use/dosage) was defined/measured/monitored (logins, logfile analysis, etc.). Use/adoption metrics are important process outcomes that should be reported in any ehealth trial.

	1	2	3	4	5	
subitem not at all important	○	○	○	○	◉	essential

Borrar selección

Does your paper address subitem 6a-ii?

Copy and paste relevant sections from manuscript text

The "3, 2, 1 Move on Study" tracked the "use" of the intervention through weekly reports. These reports helped monitor participants' adherence to the intervention schedule and progress within the platform. Additionally, a key feature of the platform was that children could not move to the next "world" or theme until they completed the full session, ensuring that each session was fully completed before progressing. This structure allowed for a clear measure of usage and engagement.

The intensity of use, or "dose," was indirectly monitored through this system, as participants had to engage with the content for the entire session before advancing. This method provided a reliable way to track how often and how thoroughly participants were interacting with the intervention, serving as an important process outcome in the study

La respuesta es demasiado larga. Prueba a acortar algunas respuestas.

6a-iii) Describe whether, how, and when qualitative feedback from participants was obtained

Describe whether, how, and when qualitative feedback from participants was obtained (e.g., through emails, feedback forms, interviews, focus groups).

	1	2	3	4	5	
subitem not at all important	○	○	○	○	◉	essential

Borrar selección

Does your paper address subitem 6a-iii?

Copy and paste relevant sections from manuscript text

Qualitative feedback from participants was obtained at the end of the project through a questionnaire. This feedback provided valuable insights into participants' experiences with the intervention, including their perspectives on the platform's usability, engagement, and effectiveness. The questionnaire allowed participants to share their thoughts on the intervention's content, the challenges they faced, and suggestions for improvement. This qualitative data helped to assess the acceptability and feasibility of the intervention and contributed to understanding the factors that influenced engagement and adherence.

6b) Any changes to trial outcomes after the trial commenced, with reasons

Does your paper address CONSORT subitem 6b? *

Copy and paste relevant sections from the manuscript (include quotes in quotation marks "like this" to indicate direct quotes from your manuscript), or elaborate on this item by providing additional information not in the ms, or briefly explain why the item is not applicable/relevant for your study

No changes were made to the trial outcomes after the trial commenced. The original outcomes outlined at the start of the study were adhered to throughout the duration of the trial without any modifications.

La respuesta es demasiado larga. Prueba a acortar algunas respuestas.

7a) How sample size was determined

NPT: When applicable, details of whether and how the clustering by care provides or centers was addressed

7a-i) Describe whether and how expected attrition was taken into account when calculating the sample size

Describe whether and how expected attrition was taken into account when calculating the sample size.

	1	2	3	4	5	
subitem not at all important	○	○	○	○	●	essential

Borrar selección

Does your paper address subitem 7a-i?

Copy and paste relevant sections from manuscript title (include quotes in quotation marks "like this" to indicate direct quotes from your manuscript), or elaborate on this item by providing additional information not in the ms, or briefly explain why the item is not applicable/relevant for your study

Tu respuesta

7b) When applicable, explanation of any interim analyses and stopping guidelines

La respuesta es demasiado larga. Prueba a acortar algunas respuestas.

Does your paper address CONSORT subitem 7b? *

Copy and paste relevant sections from the manuscript (include quotes in quotation marks "like this" to indicate direct quotes from your manuscript), or elaborate on this item by providing additional information not in the ms, or briefly explain why the item is not applicable/relevant for your study

The study did not specifically mention any interim analyses or guidelines for discontinuation during the course of the trial. In many randomized controlled trials (RCTs), interim analyses are conducted to assess whether the intervention is showing promising results or if there are any safety concerns that might require halting the trial. However, this particular study focused primarily on monitoring adherence to the intervention using weekly reports and engaging families with regular phone calls when needed, without specific mention of formal interim analyses or predefined stopping rules.

8a) Method used to generate the random allocation sequence

NPT: When applicable, how care providers were allocated to each trial group

Does your paper address CONSORT subitem 8a? *

Copy and paste relevant sections from the manuscript (include quotes in quotation marks "like this" to indicate direct quotes from your manuscript), or elaborate on this item by providing additional information not in the ms, or briefly explain why the item is not applicable/relevant for your study

"Participants will be randomly assigned in a 1:1 ratio to one of two groups: the exercise group and the routine care group. The exercise group will undergo a 12-week exercise intervention, followed by a 12-week follow-up period. On the other hand, the routine care group will undergo a 12-week period of routine care, followed by a 12-week follow-up period. If participants are not engaging 2 times per week, the research team will reach out through email and/or phone to get a clear explanation as to why and to motivate the participants to continue."

8b) Type of randomisation; details of any restriction (such as blocking and block size)

La respuesta es demasiado larga. Prueba a acortar algunas respuestas.

Does your paper address CONSORT subitem 8b? *

Copy and paste relevant sections from the manuscript (include quotes in quotation marks "like this" to indicate direct quotes from your manuscript), or elaborate on this item by providing additional information not in the ms, or briefly explain why the item is not applicable/relevant for your study

"Participants will be randomly assigned in a 1:1 ratio to one of two groups: the exercise group and the routine care group. The exercise group will undergo a 12-week exercise intervention, followed by a 12-week follow-up period. On the other hand, the routine care group will undergo a 12-week period of routine care, followed by a 12-week follow-up period. If participants are not engaging 2 times per week, the research team will reach out through email and/or phone to get a clear explanation as to why and to motivate the participants to continue."

9) Mechanism used to implement the random allocation sequence (such as sequentially numbered containers), describing any steps taken to conceal the sequence until interventions were assigned

Does your paper address CONSORT subitem 9? *

Copy and paste relevant sections from the manuscript (include quotes in quotation marks "like this" to indicate direct quotes from your manuscript), or elaborate on this item by providing additional information not in the ms, or briefly explain why the item is not applicable/relevant for your study

"Eligible participants will be randomly assigned to one of two groups using a computer-generated block randomization schedule (Research Randomizer V.4). Assignment will take place through previously constructed balanced blocks, which will allow equiprobable assignment and ensure that each group has the same number of participants. This allocation mechanism will be carried out until 84 total participants are assigned."

10) Who generated the random allocation sequence, who enrolled participants, and who assigned participants to interventions

La respuesta es demasiado larga. Prueba a acortar algunas respuestas.

Does your paper address CONSORT subitem 10? *

Copy and paste relevant sections from the manuscript (include quotes in quotation marks "like this" to indicate direct quotes from your manuscript), or elaborate on this item by providing additional information not in the ms, or briefly explain why the item is not applicable/relevant for your study

"Eligible participants will be randomly assigned to one of two groups using a computer-generated block randomization schedule (Research Randomizer V.4). Assignment will take place through previously constructed balanced blocks, which will allow equiprobable assignment and ensure that each group has the same number of participants. This allocation mechanism will be carried out until 84 total participants are assigned."

11a) If done, who was blinded after assignment to interventions (for example, participants, care providers, those assessing outcomes) and how

NPT: Whether or not administering co-interventions were blinded to group assignment

11a-i) Specify who was blinded, and who wasn't

Specify who was blinded, and who wasn't. Usually, in web-based trials it is not possible to blind the participants [1, 3] (this should be clearly acknowledged), but it may be possible to blind outcome assessors, those doing data analysis or those administering co-interventions (if any).

	1	2	3	4	5	
subitem not at all important	○	○	○	○	●	essential

Borrar selección

La respuesta es demasiado larga. Prueba a acortar algunas respuestas.

Does your paper address subitem 11a-i? *

Copy and paste relevant sections from the manuscript (include quotes in quotation marks "like this" to indicate direct quotes from your manuscript), or elaborate on this item by providing additional information not in the ms, or briefly explain why the item is not applicable/relevant for your study

"T he assignment password will be kept in a confidential f ile in the Navarrabiomed center and will be opened at the end of the study's analyses. To ensure masking, an alphanumerical code will be assigned to each study group. This code will be delivered to the associated researcher and will not be revealed to the investigators in charge of processing the data until the analysis of the coded interventions is completed. Additionally, the researchers from the Navarrabiomed center will be responsible for preparing reports for the participants' safety committee to verify the progress of the study. Following the conclusion of the baseline examination, the appropriate envelope will be opened by a study researcher, and the participants will be informed about their group allocation. Assessors of outcomes will be blinded to patient data, including allocation at baseline and follow-up. Blinding will be used for all specialized analyses. Owing to the nature of the study, patients cannot be blinded to the exercise training modality."

11a-ii) Discuss e.g., whether participants knew which intervention was the "intervention of interest" and which one was the "comparator"

Informed consent procedures (4a-ii) can create biases and certain expectations - discuss e.g., whether participants knew which intervention was the "intervention of interest" and which one was the "comparator".

	1	2	3	4	5	
subitem not at all important	○	○	○	○	◉	essential

Borrar selección

La respuesta es demasiado larga. Prueba a acortar algunas respuestas.

Does your paper address subitem 11a-ii?

Copy and paste relevant sections from the manuscript (include quotes in quotation marks "like this" to indicate direct quotes from your manuscript), or elaborate on this item by providing additional information not in the ms, or briefly explain why the item is not applicable/relevant for your study

In this study, participants were likely aware of whether they were in the exercise intervention group or not, as the intervention required active participation in physical activities through the platform. Those in the exercise group engaged with the platform's physical activity sessions, while those in the control or comparison group did not. This awareness could lead to potential biases, as participants may have developed expectations about the intervention's effects simply based on whether they were required to engage in exercises or not.

11b) If relevant, description of the similarity of interventions

(this item is usually not relevant for ehealth trials as it refers to similarity of a placebo or sham intervention to a active medication/intervention)

Does your paper address CONSORT subitem 11b? *

Copy and paste relevant sections from the manuscript (include quotes in quotation marks "like this" to indicate direct quotes from your manuscript), or elaborate on this item by providing additional information not in the ms, or briefly explain why the item is not applicable/relevant for your study

this item is usually not relevant for ehealth trials as it refers to similarity of a placebo or sham intervention to a active medication/intervention

12a) Statistical methods used to compare groups for primary and secondary outcomes

NPT: When applicable, details of whether and how the clustering by care providers or centers was addressed

La respuesta es demasiado larga. Prueba a acortar algunas respuestas.

Does your paper address CONSORT subitem 12a? *

Copy and paste relevant sections from the manuscript (include quotes in quotation marks "like this" to indicate direct quotes from your manuscript), or elaborate on this item by providing additional information not in the ms, or briefly explain why the item is not applicable/relevant for your study

"T he study will adhere to the intention-to-treat principle and use the baseline observation carried forward imputation method, where missing data will be replaced by baseline values [39]. The final analysis population will be described and any differences from the enrolled population will be reported. Participants who drop out of the study may still be included in the analysis, although their data will provide less information and lower the estimated effects variance. Adherence will be measured as the number of completed sessions divided by the total number of sessions, expressed as a percentage. The first step of the analysis will be to calculate Alonso-Martínez et al. Trials Page 10 of 13 descriptive statistics for the overall sample and between groups. Since this is an experimental design with primary and secondary outcomes measured at three timepoints - baseline (t0 = week 0), post-intervention (t1 = week 12), and after a 12-week follow-up period (t2 = week 24) - for the two study groups (exercise and routine care), a comparative analysis between the measurements will determine the differences. A linear mixed model with repeated observations will be used for these comparisons, when appropriate, for each dependent variable. Subsequently, multivariate analysis will be carried out, and the autocorrelation between repeated measures will be taken into account. We will use longitudinal analysis methods, such as a generalized estimating equation approach, to control the differences among measurements at baseline and to incorporate incomplete observations into this analysis. Pearson correlations will be calculated between the maximal values of the primary and secondary outcomes. All analyses will be 2-sided and a significance level of α ≤ 0.05 will be used for the primary analysis."

12a-i) Imputation techniques to deal with attrition / missing values

Imputation techniques to deal with attrition / missing values: Not all participants will use the intervention/comparator as intended and attrition is typically high in ehealth trials. Specify how participants who did not use the application or dropped out from the trial were treated in the statistical analysis (a complete case analysis is strongly discouraged, and simple imputation techniques such as LOCF may also be problematic [4]).

	1	2	3	4	5	
subitem not at all important	○	○	○	○	◉	essential

Borrar selección

La respuesta es demasiado larga. Prueba a acortar algunas respuestas.

Does your paper address subitem 12a-i? *

Copy and paste relevant sections from the manuscript (include quotes in quotation marks "like this" to indicate direct quotes from your manuscript), or elaborate on this item by providing additional information not in the ms, or briefly explain why the item is not applicable/relevant for your study

a completer analysis was used. This approach typically excludes data from participants who dropped out or did not complete the intervention, focusing only on those who fully participated.

12b) Methods for additional analyses, such as subgroup analyses and adjusted analyses

Does your paper address CONSORT subitem 12b? *

Copy and paste relevant sections from the manuscript (include quotes in quotation marks "like this" to indicate direct quotes from your manuscript), or elaborate on this item by providing additional information not in the ms, or briefly explain why the item is not applicable/relevant for your study

The study employed subgroup and adjusted analyses to assess the differences between the intervention (exercise group) and control group. These analyses involved comparing baseline and post-intervention metrics for physical fitness and activity levels, adjusting for potential confounders, and evaluating the statistical significance of the differences, such as changes in strength, agility, and physical activity domains. The results were examined both within each group and across the groups to understand the effect of the exercise intervention.

X26) REB/IRB Approval and Ethical Considerations [recommended as subheading under "Methods"] (not a CONSORT item)

X26-i) Comment on ethics committee approval

	1	2	3	4	5	
subitem not at all important	○	○	○	○	○	essential

La respuesta es demasiado larga. Prueba a acortar algunas respuestas.

Does your paper address subitem X26-i?

Copy and paste relevant sections from the manuscript (include quotes in quotation marks "like this" to indicate direct quotes from your manuscript), or elaborate on this item by providing additional information not in the ms, or briefly explain why the item is not applicable/relevant for your study

Tu respuesta

x26-ii) Outline informed consent procedures

Outline informed consent procedures e.g., if consent was obtained offline or online (how? Checkbox, etc.?), and what information was provided (see 4a-ii). See [6] for some items to be included in informed consent documents.

	1	2	3	4	5	
subitem not at all important	○	○	○	○	◉	essential

Borrar selección

Does your paper address subitem X26-ii?

Copy and paste relevant sections from the manuscript (include quotes in quotation marks "like this" to indicate direct quotes from your manuscript), or elaborate on this item by providing additional information not in the ms, or briefly explain why the item is not applicable/relevant for your study

The informed consent was obtained offline, in person, at the health center during a consultation with the pediatrician. The pediatrician provided participants with the necessary information about the study, explaining its purpose, procedures, and any potential risks. The consent document included a clear description of the study, how the data would be used, and the participants' rights. Families were asked to sign the consent form if they agreed to participate, ensuring that they understood the nature of the study and voluntarily consented to the use of their data.

La respuesta es demasiado larga. Prueba a acortar algunas respuestas.

X26-iii) Safety and security procedures

Safety and security procedures, incl. privacy considerations, and any steps taken to reduce the likelihood or detection of harm (e.g., education and training, availability of a hotline)

	1	2	3	4	5	
subitem not at all important	○	○	○	○	◉	essential

Borrar selección

Does your paper address subitem X26-iii?

Copy and paste relevant sections from the manuscript (include quotes in quotation marks "like this" to indicate direct quotes from your manuscript), or elaborate on this item by providing additional information not in the ms, or briefly explain why the item is not applicable/relevant for your study

The study implemented several safety and privacy protocols to protect participants and minimize potential risks. Confidentiality was ensured by storing data securely and anonymizing participant information. In terms of privacy, participants were informed about data handling procedures, including secure storage and limited access to sensitive data.

RESULTS

13a) For each group, the numbers of participants who were randomly assigned, received intended treatment, and were analysed for the primary outcome

NPT: The number of care providers or centers performing the intervention in each group and the number of patients treated by each care provider in each center

La respuesta es demasiado larga. Prueba a acortar algunas respuestas.

Does your paper address CONSORT subitem 13a? *

Copy and paste relevant sections from the manuscript (include quotes in quotation marks "like this" to indicate direct quotes from your manuscript), or elaborate on this item by providing additional information not in the ms, or briefly explain why the item is not applicable/relevant for your study

The intervention was conducted at a single healthcare center, where participants were randomly assigned, received the treatment, and were analyzed for the primary outcome.

13b) For each group, losses and exclusions after randomisation, together with reasons

Does your paper address CONSORT subitem 13b? (NOTE: Preferably, this is shown in a CONSORT flow diagram) *

Copy and paste relevant sections from the manuscript (include quotes in quotation marks "like this" to indicate direct quotes from your manuscript), or elaborate on this item by providing additional information not in the ms, or briefly explain why the item is not applicable/relevant for your study

"Eight dropouts were recorded in the exercise group because of failure to meet the minimum required sessions. One dropout occurred in the routine care group due to a change in the primary care center during the intervention."

13b-i) Attrition diagram

Strongly recommended: An attrition diagram (e.g., proportion of participants still logging in or using the intervention/comparator in each group plotted over time, similar to a survival curve) or other figures or tables demonstrating usage/dose/engagement.

	1	2	3	4	5	
subitem not at all important	○	○	○	○	○	essential

La respuesta es demasiado larga. Prueba a acortar algunas respuestas.

Does your paper address subitem 13b-i?

Copy and paste relevant sections from the manuscript or cite the figure number if applicable (include quotes in quotation marks "like this" to indicate direct quotes from your manuscript), or elaborate on this item by providing additional information not in the ms, or briefly explain why the item is not applicable/relevant for your study

Tu respuesta

14a) Dates defining the periods of recruitment and follow-up

Does your paper address CONSORT subitem 14a? *

Copy and paste relevant sections from the manuscript (include quotes in quotation marks "like this" to indicate direct quotes from your manuscript), or elaborate on this item by providing additional information not in the ms, or briefly explain why the item is not applicable/relevant for your study

"The “3, 2, 1 Move on Study” trial is a monocentric, single-blind parallel-group randomized controlled trial of 12 weeks of gamified training between November 2022 and February 2023 as is specified in Trials Clinicaltrial.gov: NCT05741879 https://clinicaltrials.gov/ct2/show/NCT05741879. The overall study procedure is illustrated in Figure 1."

14a-i) Indicate if critical “secular events” fell into the study period

Indicate if critical “secular events” fell into the study period, e.g., significant changes in Internet resources available or “changes in computer hardware or Internet delivery resources”

	1	2	3	4	5	
subitem not at all important	○	○	○	○	◉	essential

Borrar selección

La respuesta es demasiado larga. Prueba a acortar algunas respuestas.

Does your paper address subitem 14a-i?

Copy and paste relevant sections from the manuscript (include quotes in quotation marks "like this" to indicate direct quotes from your manuscript), or elaborate on this item by providing additional information not in the ms, or briefly explain why the item is not applicable/relevant for your study

No critical "secular events" occurred during the study period, such as significant changes in internet resources, hardware, or distribution infrastructure that might have impacted the intervention.

14b) Why the trial ended or was stopped (early)

Does your paper address CONSORT subitem 14b? *

Copy and paste relevant sections from the manuscript (include quotes in quotation marks "like this" to indicate direct quotes from your manuscript), or elaborate on this item by providing additional information not in the ms, or briefly explain why the item is not applicable/relevant for your study

The trial concluded after the completion of the 3-month training period for both the exercise and control groups, as initially planned and outlined in the study protocol.

15) A table showing baseline demographic and clinical characteristics for each group

NPT: When applicable, a description of care providers (case volume, qualification, expertise, etc.) and centers (volume) in each group

Does your paper address CONSORT subitem 15? *

Copy and paste relevant sections from the manuscript (include quotes in quotation marks "like this" to indicate direct quotes from your manuscript), or elaborate on this item by providing additional information not in the ms, or briefly explain why the item is not applicable/relevant for your study

The article provides a table (Table 1) showing the initial demographic and clinical characteristics of each group, which helps compare the participants in the exercise and control groups.

La respuesta es demasiado larga. Prueba a acortar algunas respuestas.

15-i) Report demographics associated with digital divide issues

In ehealth trials it is particularly important to report demographics associated with digital divide issues, such as age, education, gender, social-economic status, computer/Internet/ehealth literacy of the participants, if known.

	1	2	3	4	5	
subitem not at all important	○	○	○	○	◉	essential

Borrar selección

Does your paper address subitem 15-i? *

Copy and paste relevant sections from the manuscript (include quotes in quotation marks "like this" to indicate direct quotes from your manuscript), or elaborate on this item by providing additional information not in the ms, or briefly explain why the item is not applicable/relevant for your study

In this study, the platform was managed by the parents, who were familiar with using such platforms. This addresses key digital divide issues as the parents' familiarity with digital tools like platforms, the internet, and e-health systems played a critical role. While the study doesn't provide detailed demographic information, it can be inferred that the participants had access to technology, given the parental involvement in managing the platform. Data related to factors such as age, education, socioeconomic status, and digital literacy would be relevant to understanding potential digital barriers and ensuring broader applicability of the intervention.

16) For each group, number of participants (denominator) included in each analysis and whether the analysis was by original assigned groups

La respuesta es demasiado larga. Prueba a acortar algunas respuestas.

16-i) Report multiple “denominators” and provide definitions

Report multiple “denominators” and provide definitions: Report N’s (and effect sizes) “across a range of study participation [and use] thresholds” [1], e.g., N exposed, N consented, N used more than x times, N used more than y weeks, N participants “used” the intervention/comparator at specific pre-defined time points of interest (in absolute and relative numbers per group). Always clearly define “use” of the intervention.

	1	2	3	4	5	
subitem not at all important	○	○	○	○	◉	essential

Borrar selección

Does your paper address subitem 16-i? *

Copy and paste relevant sections from the manuscript (include quotes in quotation marks "like this" to indicate direct quotes from your manuscript), or elaborate on this item by providing additional information not in the ms, or briefly explain why the item is not applicable/relevant for your study

For the analysis, the number of participants included in each group was 32 for the intervention group and 39 for the control group, as detailed in Table 1 of the article. The analysis was conducted based on the original group assignment.

16-ii) Primary analysis should be intent-to-treat

Primary analysis should be intent-to-treat, secondary analyses could include comparing only “users”, with the appropriate caveats that this is no longer a randomized sample (see 18-i).

	1	2	3	4	5	
subitem not at all important	○	○	○	○	○	essential

La respuesta es demasiado larga. Prueba a acortar algunas respuestas.

Does your paper address subitem 16-ii?

Copy and paste relevant sections from the manuscript (include quotes in quotation marks "like this" to indicate direct quotes from your manuscript), or elaborate on this item by providing additional information not in the ms, or briefly explain why the item is not applicable/relevant for your study

Tu respuesta

17a) For each primary and secondary outcome, results for each group, and the estimated effect size and its precision (such as 95% confidence interval)

Does your paper address CONSORT subitem 17a? *

Copy and paste relevant sections from the manuscript (include quotes in quotation marks "like this" to indicate direct quotes from your manuscript), or elaborate on this item by providing additional information not in the ms, or briefly explain why the item is not applicable/relevant for your study

For each primary and secondary outcome, the results for each group and the estimated effect size along with its precision (such as the 95% confidence interval) are presented in Table 2 of the article.

17a-i) Presentation of process outcomes such as metrics of use and intensity of use

In addition to primary/secondary (clinical) outcomes, the presentation of process outcomes such as metrics of use and intensity of use (dose, exposure) and their operational definitions is critical. This does not only refer to metrics of attrition (13-b) (often a binary variable), but also to more continuous exposure metrics such as “average session length”. These must be accompanied by a technical description how a metric like a “session” is defined (e.g., timeout after idle time) [1] (report under item 6a).

	1	2	3	4	5	
subitem not at all important	○	○	○	○	○	essential

La respuesta es demasiado larga. Prueba a acortar algunas respuestas.

Does your paper address subitem 17a-i?

Copy and paste relevant sections from the manuscript (include quotes in quotation marks "like this" to indicate direct quotes from your manuscript), or elaborate on this item by providing additional information not in the ms, or briefly explain why the item is not applicable/relevant for your study

Tu respuesta

17b) For binary outcomes, presentation of both absolute and relative effect sizes is recommended

Does your paper address CONSORT subitem 17b? *

Copy and paste relevant sections from the manuscript (include quotes in quotation marks "like this" to indicate direct quotes from your manuscript), or elaborate on this item by providing additional information not in the ms, or briefly explain why the item is not applicable/relevant for your study

"Data are presented as the mean ± standard deviation. Statistical normality was determined using the Kolmogorov-Smirnov test, and significant differences were found between the groups in SLJ (cm), overall fitness, and objectively measured PA.
A paired sample t-test was used to calculate differences within each group. Additionally, a 2-sided t-test for independent samples. Moreover, Cohen's d effect sizes (ES) for differences in the studied variables between the corresponding groups (exercise group vs. routine care group; starters vs. non-starters) were calculated. Statistical analysis conducted in the study was performed using IBM SPSS version 26.0 statistical software (IBM Corporation, Armonk, NY, USA). The level of statistical significance was set at $P < 0.05$. Effect size analysis, specifically Cohen's d, was used to gauge the magnitude of differences observed both within and between groups. Cohen's d provides a standardized measure of the effect size, indicating the strength of the relationship between variables. The classification of effect sizes into small ($d=0.20–0.49$), moderate ($d= 0.50–0.79$), and large ($d≥0.80$) categories allowed for a clearer interpretation of the practical significance of the findings [37]"

18) Results of any other analyses performed, including subgroup analyses and adjusted analyses, distinguishing pre-specified from exploratory

La respuesta es demasiado larga. Prueba a acortar algunas respuestas.

Does your paper address CONSORT subitem 18? *

Copy and paste relevant sections from the manuscript (include quotes in quotation marks "like this" to indicate direct quotes from your manuscript), or elaborate on this item by providing additional information not in the ms, or briefly explain why the item is not applicable/relevant for your study

"Data are presented as the mean ± standard deviation. Statistical normality was determined using the Kolmogorov-Smirnov test, and significant differences were found between the groups in SLJ (cm), overall fitness, and objectively measured PA.
A paired sample t-test was used to calculate differences within each group. Additionally, a 2-sided t-test for independent samples. Moreover, Cohen's d effect sizes (ES) for differences in the studied variables between the corresponding groups (exercise group vs. routine care group; starters vs. non-starters) were calculated. Statistical analysis conducted in the study was performed using IBM SPSS version 26.0 statistical software (IBM Corporation, Armonk, NY, USA). The level of statistical significance was set at $P < 0.05$. Effect size analysis, specifically Cohen's d, was used to gauge the magnitude of differences observed both within and between groups. Cohen's d provides a standardized measure of the effect size, indicating the strength of the relationship between variables. The classification of effect sizes into small ($d=0.20–0.49$), moderate ($d= 0.50–0.79$), and large ($d≥0.80$) categories allowed for a clearer interpretation of the practical significance of the findings [37]"

18-i) Subgroup analysis of comparing only users

A subgroup analysis of comparing only users is not uncommon in ehealth trials, but if done, it must be stressed that this is a self-selected sample and no longer an unbiased sample from a randomized trial (see 16-iii).

	1	2	3	4	5	
subitem not at all important	○	○	○	○	○	essential

Does your paper address subitem 18-i?

Copy and paste relevant sections from the manuscript (include quotes in quotation marks "like this" to indicate direct quotes from your manuscript), or elaborate on this item by providing additional information not in the ms, or briefly explain why the item is not applicable/relevant for your study

Tu respuesta

La respuesta es demasiado larga. Prueba a acortar algunas respuestas.

19) All important harms or unintended effects in each group
(for specific guidance see CONSORT for harms)

Does your paper address CONSORT subitem 19? *

Copy and paste relevant sections from the manuscript (include quotes in quotation marks "like this" to indicate direct quotes from your manuscript), or elaborate on this item by providing additional information not in the ms, or briefly explain why the item is not applicable/relevant for your study

For the study, it was specified that any significant harms or adverse effects were primarily related to participants not following the exercise regimen. However, no major adverse effects were reported, as those who participated in the intervention were generally able to engage with the platform and follow the prescribed activities. No specific harmful events were detailed beyond the lack of adherence to the exercise program.

19-i) Include privacy breaches, technical problems

Include privacy breaches, technical problems. This does not only include physical "harm" to participants, but also incidents such as perceived or real privacy breaches [1], technical problems, and other unexpected/unintended incidents. "Unintended effects" also includes unintended positive effects [2].

	1	2	3	4	5	
subitem not at all important	○	○	○	○	○	essential

Does your paper address subitem 19-i?

Copy and paste relevant sections from the manuscript (include quotes in quotation marks "like this" to indicate direct quotes from your manuscript), or elaborate on this item by providing additional information not in the ms, or briefly explain why the item is not applicable/relevant for your study

Tu respuesta

La respuesta es demasiado larga. Prueba a acortar algunas respuestas.

19-ii) Include qualitative feedback from participants or observations from staff/researchers

Include qualitative feedback from participants or observations from staff/researchers, if available, on strengths and shortcomings of the application, especially if they point to unintended/unexpected effects or uses. This includes (if available) reasons for why people did or did not use the application as intended by the developers.

	1	2	3	4	5	
subitem not at all important	○	○	○	○	○	essential

Does your paper address subitem 19-ii?

Copy and paste relevant sections from the manuscript (include quotes in quotation marks "like this" to indicate direct quotes from your manuscript), or elaborate on this item by providing additional information not in the ms, or briefly explain why the item is not applicable/relevant for your study

Tu respuesta

DISCUSSION

22) Interpretation consistent with results, balancing benefits and harms, and considering other relevant evidence

NPT: In addition, take into account the choice of the comparator, lack of or partial blinding, and unequal expertise of care providers or centers in each group

La respuesta es demasiado larga. Prueba a acortar algunas respuestas.

22-i) Restate study questions and summarize the answers suggested by the data, starting with primary outcomes and process outcomes (use)

Restate study questions and summarize the answers suggested by the data, starting with primary outcomes and process outcomes (use).

	1	2	3	4	5	
subitem not at all important	○	○	○	○	◉	essential

Borrar selección

Does your paper address subitem 22-i? *

Copy and paste relevant sections from the manuscript (include quotes in quotation marks "like this" to indicate direct quotes from your manuscript), or elaborate on this item by providing additional information not in the ms, or briefly explain why the item is not applicable/relevant for your study

"The main findings of this study indicate that the gamified, family-based exercise intervention significantly improved adherence to 24-hour movement behavior guidelines among preschool children. Specifically, the intervention led to increased physical activity levels, reduced sedentary time, and enhanced sleep quality within the target population. Gamification was chosen to increase the likelihood of engagement and adherence in this population, based on evidence that young children respond well to interactive and playful interventions. Game mechanics, such as point accumulation, immediate rewards, and progress tracking, were included to reinforce positive behaviors and motivate consistent participation. By involving family members, the intervention also aimed to create a supportive environment that strengthens family bonds and reinforces healthy behaviors. We believe that this approach not only encourages participation but also facilitates the development of long-term physical activity habits in young children, which is essential for achieving lasting health benefits."

22-ii) Highlight unanswered new questions, suggest future research

Highlight unanswered new questions, suggest future research.

	1	2	3	4	5	
subitem not at all important	○	○	○	○	◉	essential

Borrar selección

La respuesta es demasiado larga. Prueba a acortar algunas respuestas.

Does your paper address subitem 22-ii?

Copy and paste relevant sections from the manuscript (include quotes in quotation marks "like this" to indicate direct quotes from your manuscript), or elaborate on this item by providing additional information not in the ms, or briefly explain why the item is not applicable/relevant for your study

"future research could incorporate objective tracking methods, such as wearable devices, to monitor both PA and adherence more reliably. Such tools could provide a more accurate account of daily activity and reduce dependence on parental reporting. A multi-site study design and inclusion of various PA modalities could also strengthen the generalizability of future findings."

20) Trial limitations, addressing sources of potential bias, imprecision, and, if relevant, multiplicity of analyses

20-i) Typical limitations in ehealth trials

Typical limitations in ehealth trials: Participants in ehealth trials are rarely blinded. Ehealth trials often look at a multiplicity of outcomes, increasing risk for a Type I error. Discuss biases due to non-use of the intervention/usability issues, biases through informed consent procedures, unexpected events.

	1	2	3	4	5	
subitem not at all important	○	○	○	○	◉	essential

Borrar selección

La respuesta es demasiado larga. Prueba a acortar algunas respuestas.

Does your paper address subitem 20-i? *

Copy and paste relevant sections from the manuscript (include quotes in quotation marks "like this" to indicate direct quotes from your manuscript), or elaborate on this item by providing additional information not in the ms, or briefly explain why the item is not applicable/relevant for your study

"First, the sample was drawn from a single Primary Care Center in Pamplona, which may restrict the generalizability of the results to the wider Spanish population due to potential regional variations in demographic characteristics and school environments. Future studies could consider including multiple centers across different regions to improve representativeness.
Another limitation pertains to the use of accelerometers for measuring PA. Although accelerometers provide objective data, they may not capture all types of physical activities, such as cycling, swimming, or stair climbing, potentially leading to an underestimation of overall PA levels. Additionally, the interpretation of PA intensity levels depends on predefined thresholds, which may vary by study and could influence the consistency of results across different research contexts."

21) Generalisability (external validity, applicability) of the trial findings

NPT: External validity of the trial findings according to the intervention, comparators, patients, and care providers or centers involved in the trial

21-i) Generalizability to other populations

Generalizability to other populations: In particular, discuss generalizability to a general Internet population, outside of a RCT setting, and general patient population, including applicability of the study results for other organizations

	1	2	3	4	5	
subitem not at all important	○	○	○	○	○	essential

Does your paper address subitem 21-i?

Copy and paste relevant sections from the manuscript (include quotes in quotation marks "like this" to indicate direct quotes from your manuscript), or elaborate on this item by providing additional information not in the ms, or briefly explain why the item is not applicable/relevant for your study

Tu respuesta

La respuesta es demasiado larga. Prueba a acortar algunas respuestas.

21-ii) Discuss if there were elements in the RCT that would be different in a routine application setting

Discuss if there were elements in the RCT that would be different in a routine application setting (e.g., prompts/reminders, more human involvement, training sessions or other co-interventions) and what impact the omission of these elements could have on use, adoption, or outcomes if the intervention is applied outside of a RCT setting.

	1	2	3	4	5	
subitem not at all important	○	○	○	○	○	essential

Does your paper address subitem 21-ii?

Copy and paste relevant sections from the manuscript (include quotes in quotation marks "like this" to indicate direct quotes from your manuscript), or elaborate on this item by providing additional information not in the ms, or briefly explain why the item is not applicable/relevant for your study

Tu respuesta

OTHER INFORMATION

23) Registration number and name of trial registry

Does your paper address CONSORT subitem 23? *

Copy and paste relevant sections from the manuscript (include quotes in quotation marks "like this" to indicate direct quotes from your manuscript), or elaborate on this item by providing additional information not in the ms, or briefly explain why the item is not applicable/relevant for your study

Clinicaltrial.gov: NCT05741879 https://clinicaltrials.gov/ct2/show/ NCT05741879

24) Where the full trial protocol can be accessed, if available

La respuesta es demasiado larga. Prueba a acortar algunas respuestas.

Does your paper address CONSORT subitem 24? *

Cite a Multimedia Appendix, other reference, or copy and paste relevant sections from the manuscript (include quotes in quotation marks "like this" to indicate direct quotes from your manuscript), or elaborate on this item by providing additional information not in the ms, or briefly explain why the item is not applicable/relevant for your study

Clinicaltrial.gov: NCT05741879 https://clinicaltrials.gov/ct2/show/ NCT05741879

25) Sources of funding and other support (such as supply of drugs), role of funders

Does your paper address CONSORT subitem 25? *

Copy and paste relevant sections from the manuscript (include quotes in quotation marks "like this" to indicate direct quotes from your manuscript), or elaborate on this item by providing additional information not in the ms, or briefly explain why the item is not applicable/relevant for your study

Funding for this study was provided by the Gobierno de Navarra–Departamento de Salud and co-financed by the European Regional Development Fund through the ERDF Operational Program 2014–2020 (Navarra).

X27) Conflicts of Interest (not a CONSORT item)

X27-i) State the relation of the study team towards the system being evaluated

In addition to the usual declaration of interests (financial or otherwise), also state the relation of the study team towards the system being evaluated, i.e., state if the authors/evaluators are distinct from or identical with the developers/sponsors of the intervention.

	1	2	3	4	5	
subitem not at all important	○	○	○	○	○	essential

La respuesta es demasiado larga. Prueba a acortar algunas respuestas.

Does your paper address subitem X27-i?

Copy and paste relevant sections from the manuscript (include quotes in quotation marks "like this" to indicate direct quotes from your manuscript), or elaborate on this item by providing additional information not in the ms, or briefly explain why the item is not applicable/relevant for your study

Tu respuesta

About the CONSORT EHEALTH checklist

As a result of using this checklist, did you make changes in your manuscript? *

- (x) yes, major changes
- ( ) yes, minor changes
- ( ) no

What were the most important changes you made as a result of using this checklist?

Using the checklist improved the study's clarity and transparency in several areas. It ensured the intervention's delivery was well-detailed for replication, with clear reporting of participant demographics and clinical data. The outcome analysis was enhanced by including effect sizes and confidence intervals, while safety and adverse events were better documented. The checklist also improved the reporting of informed consent procedures and ethical considerations. Overall, it helped make the study's methodology more robust and its findings more trustworthy.

How much time did you spend on going through the checklist INCLUDING making changes in your manuscript *

It took several hours over the course of a week to go through the checklist and make necessary revisions to the manuscript. This process included reviewing each section, clarifying details, and incorporating feedback to ensure all aspects of the checklist were [illegible]

La respuesta es demasiado larga. Prueba a acortar algunas respuestas.

As a result of using this checklist, do you think your manuscript has improved? *

- [x] yes
- [ ] no
- [ ] Otro:

Would you like to become involved in the CONSORT EHEALTH group?

This would involve for example becoming involved in participating in a workshop and writing an "Explanation and Elaboration" document

- [ ] yes
- [x] no
- [ ] Otro:

Borrar selección

Any other comments or questions on CONSORT EHEALTH

Tu respuesta

STOP - Save this form as PDF before you click submit

To generate a record that you filled in this form, we recommend to generate a PDF of this page (on a Mac, simply select "print" and then select "print as PDF") before you submit it.

When you submit your (revised) paper to JMIR, please upload the PDF as supplementary file.

Don't worry if some text in the textboxes is cut off, as we still have the complete information in our database. Thank you!

Final step: Click submit !

Click submit so we have your answers in our database!

La respuesta es demasiado larga. Prueba a acortar algunas respuestas.

Enviar

Borrar formulario

Nunca envíes contraseñas a través de Formularios de Google.

Este contenido no ha sido creado ni aprobado por Google. Denunciar abuso - Términos del Servicio - Política de Privacidad

Google Formularios

La respuesta es demasiado larga. Prueba a acortar algunas respuestas.

La respuesta es demasiado larga. Prueba a acortar algunas respuestas.
